# Evaluating long-term outcomes and the impact of small aortic annulus on valve replacement—a novel systematic review and meta-analysis comparing surgery vs. transcatheter interventions

**DOI:** 10.3389/fcvm.2025.1555853

**Published:** 2025-06-26

**Authors:** Aamir Amin, Cara Mohammed, Sten Kajitani, Khalid AlMashari, Rajanikant Kumar, Alifa Sabir, Paulina Briz-Echeverria, Shiva Mokhtassi, Shashi Kumar Kallikere Lakshmana, Ahmed Bokhari, Muhammad Ehsan, Hasan Ahmad, Raheel Ahmed, Toufan Bahrami

**Affiliations:** ^1^ Department of Cardiothoracic Surgery, Harefield Hospital, Guy's and St Thomas NHS Foundation Trust, London, United Kingdom; ^2^ Department of Orthopaedic Surgery, Sangre Grande Hospital, Sangre Grande, Trinidad and Tobago; ^3^ School of Medicine, University College Cork, Cork, Ireland; ^4^ College of Medicine, Imam Abdulrahman Bin Faisal University, Dammam, Saudi Arabia; ^5^ Department of CTVS, Jay Prabha Medanta Super Speciality Hospital, Patna, India; ^6^ Department of Cardiac Surgery, Rawalpindi Institute of Cardiology, Rawalpindi, Pakistan; ^7^ Department of Cardiac Surgery, Hospital Universitario de Toledo, Toledo, Spain; ^8^ Cardiothoracic Surgery, Royal Brompton Hospital, London, United Kingdom; ^9^ Department of Cardiothoracic Surgery, Jazan University, Jazan, Saudi Arabia; ^10^ Department of Medicine, King Edward Medical University, Lahore, Pakistan; ^11^ National Heart and Lung Institute, Imperial College London, London, United Kingdom; ^12^ Department of Cardiac Surgery, Royal Brompton and Harefield Hospitals, Guy's and St Thomas’ NHS Foundation Trust, London, United Kingdom

**Keywords:** transcatheter and surgical aortic valve replacement, SAVR, TAVI, small aortic annulus, TAVR—transcatheter aortic valve replacement

## Abstract

**Background:**

Transcatheter aortic valve implantation (TAVI) is often compared to surgical aortic valve replacement (SAVR) for aortic stenosis treatment. This meta-analysis evaluates the long-term efficacy and safety of TAVI vs. SAVR in aortic stenosis patients, as well as their respective impact on patients with small aortic annulus (SAA).

**Methods:**

MEDLINE, Embase, Cochrane Library, and ClinicalTrials.gov were searched for randomized controlled trials (RCTs) and comparative observational studies on TAVI vs. SAVR with long-term follow-up (3–5 years) or SAA. Risk of bias was assessed using the Cochrane Risk of Bias tool (RoB 2.0) and the Newcastle Ottawa Scale. Meta-analyses were conducted with RevMan 5.4 using a random-effects model, with risk ratio (RR) and mean difference (MD) as effect measures.

**Results:**

A total of 17 studies were included in our review. In the long-term analysis, all-cause mortality was significantly higher in the TAVI group [RR 1.10; 95% CI: 1.01–1.19], but the incidence of major bleeding [RR 0.79; 95% CI: 0.68–0.90] and atrial fibrillation was significantly lower [RR 0.37; 95% CI: 0.29–0.48] in the TAVI group. No significant difference was found between the two groups regarding other long-term outcomes. For SAA outcomes, there was no significant difference in terms of all-cause mortality [RR 0.92; 95% CI: 0.63–1.35], although cardiovascular mortality was significantly increased in the TAVI group [RR 2.08; 95% CI: 1.09–3.98]. TAVI significantly increased the rate of major vascular complications [RR 3.58; 95% CI: 1.10–11.61], aortic regurgitation/PVL [RR 6.91; 95% CI: 2.66–17.97], and pacemaker implantation (RR 2.87; 95% CI: 1.74–4.75]. TAVI significantly improved the incidence of prosthesis patient mismatch [RR 0.70; 95% CI: 0.54–0.89], effective orifice valve area (EOA) [MD 0.10; 95% CI: 0.01–0.19], and length of stay in hospital [MD −4.88; 95% CI: −5.52 to −4.23]. There were no significant differences in other clinical or echocardiographic outcomes.

**Conclusions:**

TAVI was associated with higher long-term all-cause mortality compared to SAVR in the overall population. Among patients with small aortic annulus, no survival benefit was observed with TAVI, and cardiovascular mortality was significantly increased. Future RCTs should explore SAA-related outcomes with standardized diagnostic criteria.

**Systematic Review Registration:**

https://www.crd.york.ac.uk, PROSPERO CRD42024541862.

## Introduction

Inarguably, aortic stenosis (AS) is one of the most prevalent valvular heart diseases in the elderly population that is defined by a progressively narrowed aortic valve orifice (1). The causative leaflet calcification restricts normal blood flow to the aorta resulting in a hypertrophied left ventricle and, if left untreated, may lead to heart failure and death (2, 3). The latest American College of Cardiologists (ACC) and American Heart Association (AHA) guidelines recommend surgical aortic valve replacement (SAVR) as the standard treatment for AS patients with low to moderate surgical risk and a higher life expectancy, thus offering a definitive remedy with enduring valve function (4). SAVR involves either a traditional open-heart surgical approach or minimally invasive techniques in which one or more chest incisions are made to access the heart and replace the stenosed valve (5). However, the invasive nature of the procedure and the associated prolonged recovery time has led to alternative treatments with potentially less impact on patient quality of life (5). Moreover, in the small aortic annulus (SAA) demographic which is generally defined by either not fitting a >21 mm surgical valve or having an annular size ≤400–430 mm (2) *via* echocardiographic imaging or direct sizing, SAVR is reportedly associated with a higher incidence of suboptimal hemodynamic and clinical outcomes such as increased risk of patient-prosthesis mismatch (PPM) (6–8). In SAVR, a small annulus may necessitate additional techniques like annular enlargement to accommodate a standard-sized valve, therefore increasing peri-procedural complexity and risks that include but are not limited to serious complications like annular rupture, thus potentially extending patient recovery time (9).

Over the past two decades, transcatheter aortic valve implantation (TAVI) has been introduced as a formidable interventional alternative to SAVR, which facilitates the replacement of a stenosed valve without surgical removal. TAVI is typically performed by inserting a catheter device with either a self- or balloon-expandable valve prosthesis *via* the femoral approach. However, alternate access routes are considered when necessary with the goal of pushing aside the damaged leaflet and taking over its function. Several randomized controlled trials (RCTs) have illustrated that TAVI is a non-inferior procedure, compared to SAVR for inoperable or intermediate to high-risk aortic stenosis patients in terms of all-cause mortality and stroke (10–16). Superiority, especially for SAA patients as regards PPM and duration of hospital stay was also reported in some RCTs and observational studies (12, 17–19). However, an increased risk of moderate to severe paravalvular leakage (PVL) and new permanent pacemaker implantation have persisted as concerns surrounding its use (12–14, 17–40).

Although multiple systematic reviews and meta-analyses comparing the clinico-echocardiographic outcomes for both interventions among AS patients have been published (20–23), most of them are limited by the inclusion of short-term (1–2 years) pooled results, thus potentially overlooking crucial long-term data (24). In addition, 3–5 year outcomes of the PARTNER 3 and EVOLUT Low-Risk trials have recently been published which have not yet been incorporated into any meta-analysis (25, 26). Furthermore, till date no comprehensive review of the existing literature has been undertaken to evaluate the clinical and echocardiographic outcomes associated with SAA in aortic stenosis patients undergoing SAVR or TAVI.

This systematic review and meta-analysis aims to update the current evidence base regarding the long-term efficacy and safety of SAVR and TAVI as treatment modalities for patients with AS and address knowledge gaps regarding the clinical implications of both procedures in patients with SAA.

## Material and methods

This review has been registered with the International Prospective Register of Systematic Reviews (PROSPERO) under the identifier CRD42024541862. This study was performed following the recommendations of the Cochrane Handbook for Systematic Reviews of Interventions (27) and reported according to the Preferred Reporting Items for Systematic Reviews and Meta-Analysis (PRISMA) statement (28). Ethical approval was not required for this study.

### Eligibility criteria

The inclusion criteria were as follows:

1) Population: Patients with aortic valve stenosis with or without small aortic annulus.
2) Intervention: Transcatheter Aortic Valve Replacement/Implantation (TAVI)
3) Comparator: Surgical Aortic Valve Replacement (SAVR)
4) Outcome: reporting at least 1 outcome of interest (long-term outcomes are to be assessed at a 3–5-year follow-up period)
5) Study design: Randomized Controlled Trials (RCTs) (RCTs only for long-term outcomes) and comparative observational studies.

Exclusion Criteria:

1) Patients with valvular heart disease other than aortic valve stenosis.
2) Non-comparative studies including patients who had either TAVI only or SAVR only but not both.
3) All other study designs apart from those listed in both inclusion criteria such as case studies, case series, and quasi-randomized trials.
4) Studies that assess short-term outcomes (<3 years) in patients with aortic stenosis without SAA.

### Information sources

We searched the following electronic databases and international trial registers from inception to May 2024 with no language restrictions: the Cochrane Central Register of Controlled Trials (CENTRAL, *via* The Cochrane Library), MEDLINE (*via* PubMed), Embase (*via* Ovid), and ClinicalTrials.gov. We also explored grey literature sources such as ProQuest Dissertations and Theses Global (PQDT) and OpenGrey to identify additional relevant studies. The reference lists of included articles and relevant systematic reviews were screened to find other potentially eligible studies. We also performed forward citation tracking using the Web of Science to retrieve any other potential studies.

We used a search strategy with keywords and Medical Subject Headings (MeSH) terms pertaining to aortic valve stenosis, transcatheter aortic valve implantation, transcatheter aortic valve replacement, and surgical aortic valve replacement. The detailed search strategy is given in Supplementary Table S1.

### Selection process

We imported all the articles retrieved through our search process into Rayyan for deduplicating and screening. Following deduplication, two authors independently conducted the initial screening phase, evaluating titles and abstracts. The same authors performed the subsequent full-text screening on the remaining articles. Any conflict was resolved by a third reviewer.

### Data collection process and data items

Following the study selection process, two reviewers extracted data into a piloted structured Excel spreadsheet to maintain consistency. Essential data items were then meticulously collected including study characteristics (trial name, location, recruitment dates, study arms, number of patients, follow-up duration) baseline patient characteristics (age, sex, comorbid conditions, NYHA class, mean aortic annulus diameter, minimal aortic annulus diameter, aortic annulus area, LVEF, mean aortic gradient, maximal aortic gradient, aortic valve area), intervention characteristics (TAVI route, valve name, Edward SAPIEN valve type) and outcome measures.

### Outcome measures

Our primary outcomes were all-cause mortality and stroke. Secondary outcomes included cardiovascular mortality, disabling stroke, MI, major bleeding, new PPM implantation, atrial fibrillation (new or worsened), AKI, endocarditis, reintervention, rehospitalization, major vascular complications, death or disabling stroke, death, stroke or hospitalization, length of stay in ICU, length of stay in hospital, effective orifice/valve area, valve/orifice area index, aortic regurgitation/PVL, patient-prosthesis mismatch and mean valve gradient. Long-term outcomes were assessed at a 3–5 year follow-up.

### Risk of bias assessment

We evaluated the risk of bias in the included RCTs using the revised Cochrane Risk of Bias tool for randomized trials (RoB 2.0), which assesses bias in the following 5 domains: (1) bias arising from the randomization process; (2) bias caused by deviations from intended interventions; (3) bias caused by missing outcome data; (4) bias in the measurement of the outcome, and (5) bias in the selection of the reported result.

The methodological quality of observational studies was assessed independently using Newcastle Ottawa Scale (NOS) for cohort studies. Studies were allocated stars based on three perspectives: the selection of the study groups; the comparability of the groups; and the ascertainment of the outcome of interest. Two review authors independently applied the tools to each included study. Any disagreements were resolved by discussion to reach a consensus between the two review authors. If the matter remained unresolved, a third review author acted as a judge to give a final decision.

### Data synthesis

We performed meta-analyses using Review Manager (RevMan, version 5.4; The Cochrane Collaboration, Copenhagen, Denmark). Dichotomous outcomes were presented as relative risk (RR) with 95% confidence intervals (CIs) and continuous outcomes were pooled as mean difference (MD) with 95% confidence intervals (CIs). To ensure consistency in our analyses, we transformed medians and interquartile ranges (IQRs) into means and standard deviations (SDs). The Der Simonian and Laird random-effects model was used to perform meta-analyses. Publication bias was planned to be assessed using a funnel plot if there were at least 10 studies present in a synthesis. The asymmetry of the funnel plot was checked using Egger's regression test. For each synthesis, the *I*^2^ index and the chi-square test were used for the assessment of heterogeneity, and a *P* value of 0.1 was considered critical for the heterogeneity of the included studies. We will interpret *I*^2^ values according to the Cochrane Handbook for Systematic Reviews of Interventions, section 10.10.

## Results

### Study selection

**Figure 1:**
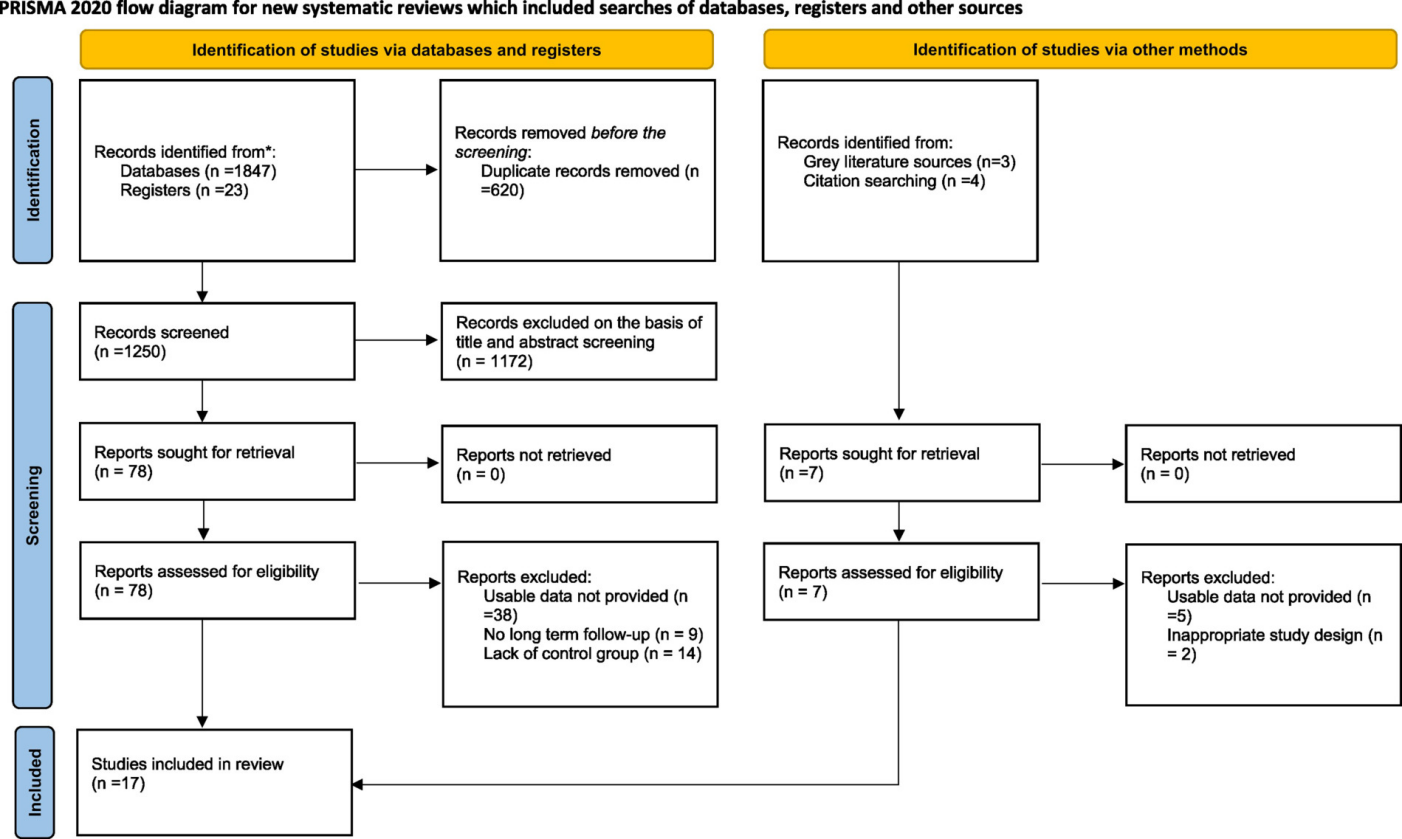
PRISMA 2020 flow chart of included and excluded trials. PRISMA, preferred reporting items for systematic reviews and meta-analyses.

A total of 1,670 studies were retrieved from our database search. Furthermore, 7 number of studies were obtained from grey literature sources. A total of 620 duplicates were removed before the screening process. After screening, 17 articles were included in this systematic review and meta-analysis. The detailed selection process is illustrated using a PRISMA Flowchart (Figure 1).

### Study characteristics

A total of 1,024 patients were included in the seventeen studies included in this systematic review and meta-analysis. In the TAVI group, 489 patients were included whereas 535 patients were included in the SAVR group. The mean age was comparable between the TAVI and SAVR groups, with only four studies reporting an age difference of more than 5 years. The transfemoral route was the most common route employed for TAVI in most (70.6%) of the studies (*n* = 12). The follow-up period of the included studies ranged from 1 year–5 years. The detailed study characteristics are presented in Table 1. Regarding NYHA Functional Class III or IV, the mean number in TAVI was 281.6 ± 248.8 whereas in SAVR, it was 272.5 ± 248.6. Supplementary Table S2 shows the clinical characteristics and outcomes of the included studies.

**Table 1:** Study characteristics of the included studies.

Study authors	No. of patients			Age (years)		Male (%)		TAVI route	Valve name	Edwards SAPIEN valve (Edwards Lifesciences, Irvine, CA)	Follow up
	Total	TAVR	SAVR	TAVR	SAVR	TAVR	SAVR				
Gleason et al. (10)	744	390	354	83.2 ± 7.1	83.3 ± 6.4	52.9	52.4	Transfemoral and non-TF	CoreValve vs. valve selection left at surgeon's discretion	TAVR: Self-Expanding. SAVR: biological valve (98.6%) and the remaining received a mechanical valve (1.4%)	5 years
Deeb et al. (40)	744	390	354	83.2 ± 7.1	83.3 ± 6.4	52.9	52.4	Transfemoral and non-TF	CoreValve vs. valve selection left at surgeon's discretion	TAVR: Self-Expanding. SAVR: biological valve (98.6%) and the remaining received a mechanical valve (1.4%)	2 years
Mack et al. (25)	950	496	454	73.3 ± 5.8	73.6 ± 6.1	67.5	71.1	Transfemoral	(Edwards SAPIEN 3) vs. (Carpentier Edwards Perimount, Magna, Magna Ease, Intuity, Intuity Elite, Mosaic, Mosaic Ultra, Freestyle, Hancock II, Trifecta, Epic, Epic Ultra, Perceval, Perceval S, Crown PRT, Mitroflow, and other unknown types)	(Balloon-expandable) vs. (Stented and stentless bioprostheses)	5 years
Kamioka et al. (19)	94	35	59	85.1 ± 6.0	77.4 ± 8.7	8.6	5.1	Transapical	(Edwards Sapien XT 23-mm) vs. (Carpentier-Edwards prosthesis Magna Ease, Mosaic Ultra)	(Ballon-expandable) vs. (Stented bioprostheses)	2 years
Forrest et al. (26)	1414	730	684	74.1 ± 5.8	73.7 ± 5.9	63.6	65.9	-	TAVI—CoreValve, Evolut R, or Evolut PRO, Medtronic	self-expanding, supra-annular valve	3 years
Clavel et al. (17)	150	50	100	83 ± 7	75 ± 6	54	54	Transfemoral (76%) and transapical (24%)	TAVI—Cribier-Edwards or Edwards SAPIEN (Edwards Lifesciences Inc, Irvine, Calif) vs. SAVR—Edwards Perimount Magna or (Medtronic Freestyle, Medtronic, Minneapolis, Minnesota	balloon-expandable prostheses vs. stented bioprosthesis (71%) and mechanical valve (29%)	1 year
Makkar et al. (13)	2032	1,550	482	81.5 ± 6.7	81.7 ± 6.7	54.2	54.8	Transfemoral or transthoracic	(Edwards Sapien XT) vs. (Edwards lifesciences surgical bioprosthesis)	Balloon-expandable vs. stented bioprosthesis	5 years
Nishigawa et al. (42)	277	214	63	84.7 ± 4.8	76.4 ± 5.3	19.2	21.6	Transfemoral or transapical	(Edwards Sapien XT or Sapien 3) vs. (Edwards Magna Ease or Inspiris)	Balloon-expandable vs. stented bioprosthesis	1-yr
Van Meighem et al. (14)	1,660	864	796	79.8 ± 6.2	79.7 ± 6.1	57.6	55	Transfemoral	TAVR group under- went implant of a first-generation (CoreValve; Medtronic) or second-generation (Evolut R valve; Medtronic)|surgical valve type was per operator's choice, although mechanical valves were not allowed.	Self-Expandings vs Not specified	5 years
Rodes-Cabau et al. (16)	151	77	74	75.9 ± 5.3	75.1 ± 4.9	5.2	9.5	Transfemoral 69/76 (90.8%) -Transcarotid/transaortic 7/76 (9.2%)	TAVR: balloon-expandable SAPIEN 3/Ultra valve (Edwards Lifesciences, Irvine, CA), the self-expandable Evolut R/PRO/PRO+/FX valve (Medtronic, Minneapolis, MN), and the Acurate neo/neo2 valve (Boston Scientific, Boston, MA) | The SAVR procedures were performed according to the standards of the surgical team of each participating center, and all surgical prosthetic valves approved for clinical use were allowed in the study.	Balloon-expandable 31/76 (40.8%) Self-expandable 45/76 (59.2%)	4 years
Dionne et al. 2017	163	50	113	83.1 ± 7	79.4 ± 5.6	-	-	Transfemoral, trans apical, transaortic	SAPIEN-XT bioprosthesis (Edwards lifesciences Corp, Irvine, CA USA) vs Sorin Perceval bioprosthesis (Sorin, Saluggia, Italy). before 2012 SAPIEN THV.	Balloon expandable	-
Repossini et al. (38)	284	142	142	76.2 ± 7.6	76.4 ± 7.2	59.9	61.9	-	-	-	-
Guimaraes et al. (18)	714	357	357	80 ± 8	74 ± 9	20	20	Transesophageal/transthoracic	TAVR—balloon expandable valves: Edwards SAPIEN, SAPIEN XT, and SAPIEN 3. Self expandable valves: Corevalve (Medtronic), EVOLUT R (Medtronic), Engager (Medtronic), Portico (St. Jude Medical), Acurate (Boston Scientific) and HLT Meridian (HLT). SAVR—Stented valvles: Mitro-flow (Sorin), Mosaic (Medtronic), Epic (St Jude Medical), Perimount Magna Ease Pericardial (Carpentier—Edwards) and Trifecta (St Jude Medical). Stentless valves: Solo—Freedom (Sorin). Sutureless valves: Perceval (Sorin) and Enable 3f (Medtronic).	TAVR—balloon expandable valves and self expandable valves. SAVR—stented valves, stentless valves and sutureless valves.	-
Mack et al. (25)	699	348	351	84.1 ± 6.6	-	-	-	Transfemoral, transapical	-	-	5 years
Thyregod et al. (33)2019	280	145	135	79.2 ± 4.9	79.0 ± 4.7	53.8	52.6	-	-	-	
Rodes-Cabau et al. (16)	574	304	270	84 ± 6	85 ± 6	41	33	Transfemoral and transapical	balloon-expandable Edwards SAPIEN valve (Edwards Lifesciences, Irvine, CA)	Balloon-expandable	2 years
Salna 2018	170	40	130	84	82	2	9	Transfemoral and transaortic	Carpentier-Edwards Magnaa, Medtronic Mosaicb, Medtronic Corevalve Evolutb, Edwards Sapien 3a	Bovine and procine Pericardial xenografts vs stented bovine and porcine pericardial xenografts	14 months vs 28 months

### Quality assessment of the included studies

Most of the included RCTs (5/8) were assessed to have a high risk of bias due to missing outcome data, non-blinding, and lack of an appropriate analysis method. All the studies had some risk of bias in the domain of randomization due to problems with the randomization process and a lack of information about allocation concealment (Supplementary Figure S1).

NOS was used for the assessment of the risk of bias in nine observational studies. Most of the studies (5/9, 56%) had a low risk of bias, 3 studies had a medium risk of bias, while only 1 study was assessed to have a high risk of bias. [Supplementary Table S3].

### Outcomes for TAVI vs. SAVR

#### All-cause & cardiovascular mortality

**Figure 2:**
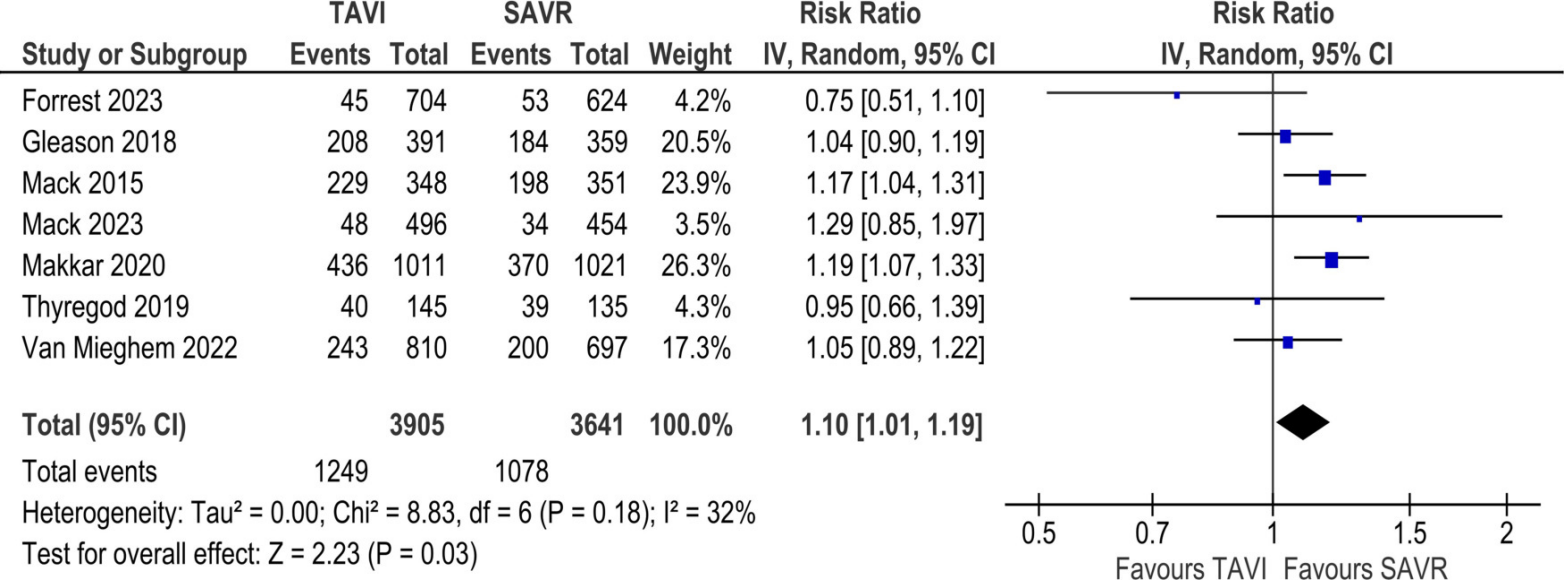
Incidence of all-cause mortality at 3 to 5-year follow-up (TAVI vs. SAVR).

In the long-term analysis, the risk of all-cause mortality was significantly increased in the TAVI group as compared to the SAVR group (RR 1.10; 95% CI: 1.01–1.19; Figure 2). Statistical heterogeneity was found to be moderate (*I*^2^ = 32%). However, no statistically significant difference was found between the two groups regarding cardiovascular mortality [RR 1.08; 95% CI (0.82–1.28), *I*^2^ = 0%; Supplementary Figure S2].

**Figure 3:**
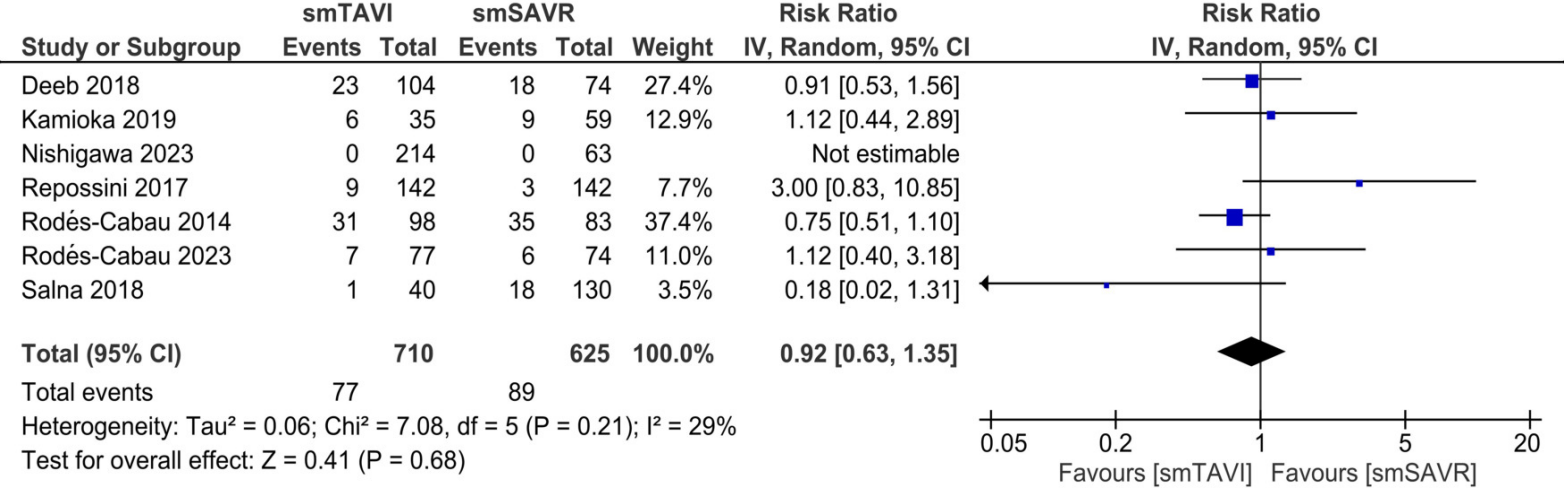
Incidence of all-cause mortality in patients with SAA (TAVI vs. SAVR).

In the SAA analysis, there was no statistically significant difference in terms of all-cause mortality between the TAVI and SAVR groups [RR 0.92; 95% CI (0.63–1.35), *I*^2^ = 29%; Figure 3]. However, the risk of mortality from cardiovascular causes was significantly increased in the TAVI group as compared to the SAVR group [RR 2.08; 95% CI (1.09–3.98); Supplementary Figure S14]. Heterogeneity was found to be mild (*I*^2^ = 17%).

#### Total & disabling stroke

**Figure 4:**
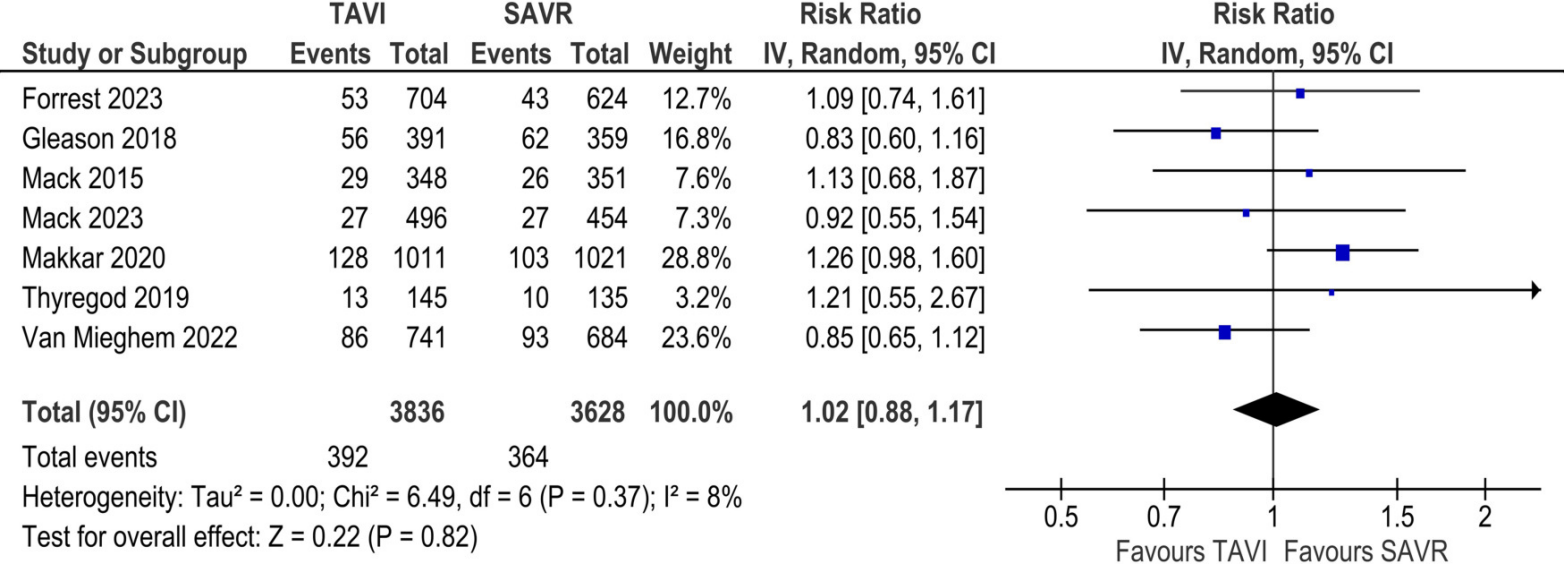
Incidence of stroke at 3 to 5-year follow-up (TAVI vs. SAVR).

In the long-term analysis, there was no statistically significant difference between the TAVI and SAVR groups concerning the incidence of stroke [RR 1.02; 95% CI (0.88–1.17), *I*^2^ = 8%; Figure 4] and disabling stroke [RR 0.92; 95% CI (0.75–1.14), *I*^2^ = 6%; Supplementary Figure S3].

**Figure 5:**
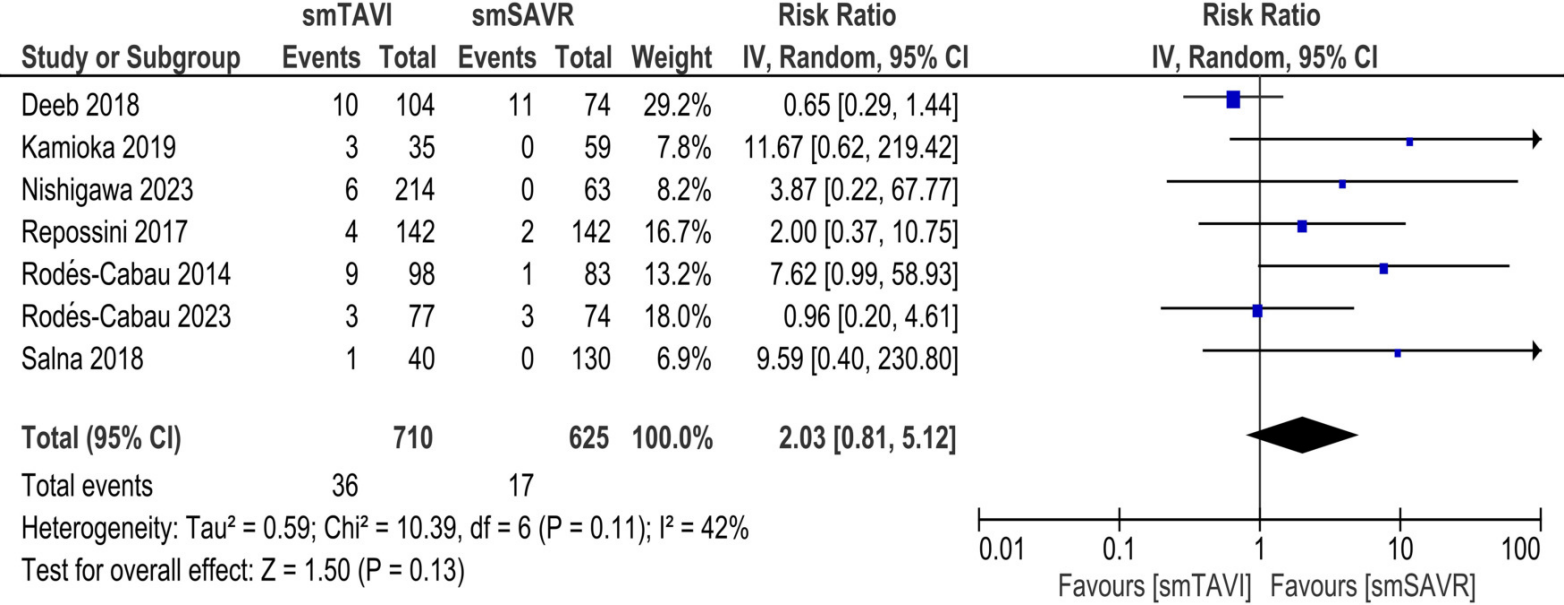
Incidence of stroke in patients with SAA (TAVI vs. SAVR).

In the SAA analysis, there was no statistically significant difference between the TAVI and SAVR arms with respect to the incidence of stroke [RR 2.03; 95% CI (0.81–5.12), *I*^2^ = 42%; Figure 5] and disabling stroke [RR-0.78; 95% CI (0.26–2.34), *I*^2^ = 0%; Supplementary Figure S15].

#### Death or disabling stroke

In the long-term analysis, no statistically significant difference was found between the two groups regarding the composite outcome of death or disabling stroke [RR 1.08; 95% CI (0.98–1.19); Supplementary Figure S4]. The interstudy heterogeneity was estimated to be moderate (*I*^2^ = 53%).

In the SAA analysis, no statistically significant difference was found between the two groups regarding the composite outcome of death or disabling stroke [RR 1.08; 95% CI (0.57–2.03); Supplementary Figure S16] The interstudy heterogeneity was estimated to be moderate (*I*^2^ = 39%).

#### Death, stroke or hospitalization

In the long-term analysis, no statistically significant difference was found between the two groups regarding the composite outcome of death, stroke, or hospitalization [RR 1.01; 95% CI (0.85–1.19); Supplementary Figure S5]. Statistical heterogeneity was estimated to be substantial (*I*^2^ = 79%). In the SAA analysis, no statistically significant difference was found between the two groups regarding the composite outcome of death, stroke, or hospitalization [RR 1.43; 95% CI (0.47–4.37); Supplementary Figure S17]. Statistical heterogeneity was estimated to be substantial (*I*^2^ = 63%).

### Secondary peri-operative outcomes

Perioperative outcomes were measured according to the VARC endpoint definitions (29). In the long-term analysis, the incidence of major vascular complications was not significantly different between the TAVI and SAVR arms [RR 2.15; 95% CI (0.99–4.70); Supplementary Figure S6] with substantial heterogeneity (*I*^2^ = 78%). In contrast, the major bleeding rate was significantly higher in the SAVR arm than in the TAVI [RR 0.79; 95% CI (0.68–0.90), *I*^2^ = 0%; Supplementary Figure S7].

No statistically significant difference was found between the two groups regarding the incidences of myocardial infarction [RR 1.05; 95% CI (0.79–1.40), *I*^2^ = 34%; Supplementary Figure S8] or infective endocarditis [RR 0.85; 95% CI (0.53–1.37), *I*^2^ = 42%; Supplementary Figure S9].

TAVI significantly decreased the rate of atrial fibrillation [RR 0.37; 95% CI (0.29–0.48); Supplementary Figure S10]. The inter-study heterogeneity was calculated to be substantial (*I*^2^ = 76%). Conversely, re-hospitalization [RR 1.09; 95% CI (0.89–1.35), *I*^2^ = 74%; Supplementary Figure S11] and re-intervention rates [RR 1.48; 95% CI (0.98–2.25), *I*^2^ = 0%; Supplementary Figure S12] were not significantly different between the two arms. TAVI significantly increased the rate of permanent pacemaker implantation compared to SAVR [RR 1.96; 95% CI (1.43–2.69); Supplementary Figure S13]. The inter-study heterogeneity was estimated to be substantial (*I*^2^ = 86%).

In the SAA analysis, TAVI significantly increased the rate of major vascular complications as compared to SAVR [RR 3.58; 95% CI (1.10–11.61); Supplementary Figure S18] with moderate heterogeneity (*I*^2^ = 30%). In contrast, the incidence of major bleeding was statistically non-significant between the two arms [RR 0.91; 95% CI (0.57–1.45), *I*^2^ = 79%; Supplementary Figure S19].

No statistically significant difference was found between the two groups regarding the incidence of myocardial infarction [RR 0.46; 95% CI (0.10–2.09), *I*^2^ = 0%; Supplementary Figure S20] or LOS in the ICU [MD 0.75; 95% CI (−0.98–2.49), *I*^2^ = 92%; Supplementary Figure S21]. In contrast, the LOS in the hospital was statistically significant, favoring TAVI [MD −4.88; 95% CI (−5.52 to −4.23), *I*^2^ = 14%; Supplementary Figure S22].

Conversely, re-hospitalization [RR 1.09; 95% CI (0.89–1.35), *I*^2^ = 74%; Supplementary Figure S23] and re-intervention rates [RR 1.48; 95% CI (0.98–2.25), *I*^2^ = 0%; Supplementary Figure S24] were not significantly different between the two arms.

## Echocardiography outcomes

In the SAA analysis, the rate of PPM (moderate/severe) was significantly decreased in the TAVI group [RR 0.70; 95% CI (0.54–0.89); Supplementary Figure S25]. Heterogeneity was estimated to be substantial (*I*^2^ = 58%). The rate of pacemaker implantation was found to be significantly increased in the TAVI group [RR 2.87; 95% CI (1.74–4.75), *I*^2^ = 0%; Supplementary Figure S26].

TAVI significantly improved the effective orifice area (EOA) compared to SAVR [MD 0.10; 95% CI (0.01–0.19); Supplementary Figure S27] with considerable heterogeneity (*I*^2^ = 80%). However, no significant difference was observed between the two groups regarding the EOAI [MD 0.06; 95% CI (−0.01–0.13), *I*^2^ = 85%; Supplementary Figure S28]. The rate of PVL was significantly increased in the TAVI group [RR 6.91; 95% CI (2.66–17.97), *I*^2^ = 0%; Supplementary Figure S29].

## Discussion

This is a unique, two-pronged systematic review and meta-analysis comparing both long-term and SAA-related outcomes for patients undergoing TAVI vs. SAVR. The 2021 European Society of Cardiology/European Association for Cardiac and Thoracic Surgery (ESC/EACTS) guidelines on the management of severe aortic stenosis use thresholds of age <75 years and low surgical risk to recommend SAVR and age ≥75 years for TAVI, while the 2020 American College of Cardiology/American Heart Association (ACC/AHA) guidelines use age thresholds of <65 years or life expectancy >20 years to recommend SAVR and age >80 years or life expectancy <10 years to recommend TAVI (30). Moreover, the use of stentless valves and aortic root enlargement have been proposed as strategies for SAA management in the ESC/EACTS guidelines (31). However, there have been no updates to these international guidelines over the past three years.

In our long-term analysis, we found that all-cause mortality and the need for PPM implantation increased significantly with the use of TAVI compared to SAVR. Conversely, SAVR was demonstrably inferior to TAVI in the risk for major bleeding and new-onset atrial fibrillation at 3–5-year follow-up. Other outcomes showed no statistically significant long-term variations between the two groups, thus demonstrating comparable risk and/or benefit. For SAA-related outcomes, TAVI was associated with a significantly reduced risk of cardiovascular mortality and a lesser duration of hospital LOS. Our meta-analysis also yielded results favoring SAVR in the risk for major vascular complications, PPM implantation and PVL, that were significantly higher for SAA-related outcomes in the TAVI group. Furthermore, our analysis of long-term echocardiographic findings revealed an increased risk of moderate to severe PPM with TAVI compared to SAVR. Although EOA was significantly larger in the TAVI group, EOAI—a parameter that adjusts for body surface area and better reflects clinical relevance—did not differ significantly. This suggests that the anatomical design advantage of TAVI may not translate into proportional functional benefit in all patients. Hence, EOAI should be prioritized when evaluating prosthetic performance. Analysis of the remaining outcomes revealed no significant difference between the two groups.

Our study refutes both the superiority and non-inferiority of TAVI to SAVR in the risk for all-cause mortality as reported by previous systematic reviews and meta-analyses (21, 32–36). Moreover, we observed no significant difference in the incidence of major vascular complications following TAVI which was observed to be elevated in comparison to SAVR by several former appraisals (20, 33, 34, 36).

Nonetheless, our analysis of long-term findings is consistent with earlier reviews in demonstrating the superiority of SAVR in the risk of PPM implantation (3–6, 9), moderate to severe PPM (32) and PVL (32, 33, 36), which is reportedly greater following TAVI, excluding increased PPM incidence among high-risk surgical patients (37). We also found that TAVI was superior to SAVR with respect to the incidence of new-onset atrial fibrillation (20, 34, 36), major bleeding (20, 34, 36), and hospital LOS (20) in congruence with previous studies.

We observed no significant difference in the total stroke rate, the risk of disabling strokes, composite outcomes for death or disabling stroke, and the composite outcomes of death, stroke, or re-hospitalization between TAVI and SAVR like other previous systematic reviews and meta-analyses, unlike two studies that reported increased risk of stroke after TAVI (33, 36) and two others reported higher re-hospitalization rates following TAVI at 2-year follow-up (20, 21). This comparability further extends to the risk of MI (36), infective endocarditis (36) and ICU LOS, except one study which reported an increased risk of MI with TAVI (34) and a shorter ICU LOS with TAVI (20).

Although a greater risk of mortality was reported among SAA patients following TAVI than SAVR at 30-days follow-up by one study (38) and a lower risk by PARTNER 2 (39), we found no statistical difference between the two in conjunction with the findings of other recent studies (16, 19, 40). Moreover, TAVI was associated with a significantly decreased risk of cardiovascular mortality than SAVR in our review as opposed to a previous study reporting no significant difference between them (40). Cardiovascular mortality differences may reflect variations in baseline patient characteristics, procedural expertise, and post-procedural care across studies. Additionally, inconsistencies in endpoint definitions and adjudication methods may contribute to outcome variability. Extreme myocardial hypertrophy, frequently observed in patients with SAA, may exacerbate PPM and impair post-procedure remodelling. Severe LVH is poor prognostic factor after TAVR, potentially contributing to increased cardiovascular mortality (41).

We further observed that TAVI was inferior to SAVR in terms of major vascular complications (38, 40), the risk of PVL (38, 40, 42) and PPM implantation (38, 40), which is consistent with our findings in the long-term analysis of outcomes between TAVI vs. SAVR and prior studies (16, 42). Although two studies reported a similar risk of PPM among SAA patients (16, 42) in the TAVI and SAVR groups, our findings were consistent with two studies that reported a lower incidence among TAVI patients (17, 40). Moreover, in contrast to the long-term outcomes analysis and data reported by a recent study (40), we observed that the risk for major bleeding was similar in both groups.

The remaining endpoints such as the risk of MI, infective endocarditis, the total stroke rate, the risk of disabling strokes, composite outcomes for death or disabling stroke, and the composite outcomes of death, stroke, or hospitalization were comparable between TAVI and SAVR for SAA between our study and most previous reports (16, 38, 40, 42), as was the length of ICU stay and we report no significant difference between them. It is noteworthy that patient age as a baseline characteristic was well-balanced in most of the included RCTs (10, 13, 25, 26).

A critical concern, especially in younger populations, is structural valve deterioration (SVD) associated with valve replacement procedures. Limited follow-up in many studies precludes robust conclusions about SVD rates. A recent study found low rate of SVD with the use of self-expanding TAVR compared to SAVR (43). However, extended follow-up data is needed to compare valve longevity.

As the first systematic review and meta-analysis evaluating the impact of SAA on TAVI vs. SAVR, our study reports data on previously unexplored outcomes. Although long-term outcomes have been evaluated between the two groups in a prior study, it included data published at 1–2 year follow-up from ongoing trials (21). In comparison, we implemented a robust search strategy across databases using stringent criteria which excluded all trials except those reporting data at 3–5 years follow-up (10, 11, 13, 14, 25, 26, 44). Previous appraisals pooled outcomes for the two groups and included studies on elderly patients with greater disease severity and, therefore, a disproportionately higher peri-operative risk (21, 35–36). Other studies also included patients with low to intermediate surgical risk leading to heterogeneity and confounding the results (35, 36). Despite these restrictions, our study reports data from a larger sample size and incorporates data from the most recent RCTs comparing TAVI vs. SAVR, including the Mack and Forrest trials (25, 26).

Our study is influenced by certain limitations. We were unable to conduct a head-to-head, individual-level patient data analysis between the TAVI and SAVR groups for either the long-term follow-up or the SAA outcomes comparison. Intention-to-treat was not performed in the included studies and a lack of standardization among the definitions for post-procedural outcomes were notable limitations (10, 11, 13, 14, 25, 26, 44). The severity of aortic regurgitation, for example, was not specified by each of the included studies. There were considerable differences across study methodology and follow-up durations (10, 11, 13, 14, 16–19, 25, 26, 40, 42, 44). Moreover, due to a lack of information on the exact interventional or operative process, we were unable to compare differences between various routes and approaches that could contribute to differences in outcomes as reported in a previous review (32). For the SAA comparison, the diagnostic criteria of SAA are dissimilar in the included literature and there is no universally accepted definition (16–19, 38, 40, 42). The inclusion of observational studies due to the lack of RCTs (16–19, 38, 40, 42) on the topic was a prominent limitation and could potentially contribute to increased heterogenicity and confounding bias. Furthermore, heterogeneity across outcomes such as LOS, EOA, and PPM implantation limits the precision of pooled estimates and highlights the need for homogenous endpoints across future studies. Lastly, we could not perform subgroup analysis based on valve morphology (tricuspid or bicuspid), which could have significantly impacted the results.

Our study highlights several new implications for research and clinical practice involving the choice of intervention for aortic valve disease, hence calling for updates to be made in the present international guidelines on its management (30, 31). Furthermore, the evaluation of SAA-related outcomes between the two groups requires a randomized approach to better inform clinical decision-making (16–19, 38, 40, 42). There is a need for RCTs comparing different TAVI routes to SAVR that have been shown to affect patient outcomes (10, 11, 13, 14, 25, 26, 44). A standardisation of SAA definitions to create a universal understanding can help improve disease classification for research and create a wider understanding of the best practice.

The increased long-term risk of mortality with TAVI observed in our study is an important implication for cardiologists and cardiac surgeons. Additionally, 3–5-year follow-ups inch closer to helping establish a more definitively stratified risk and complication profile for TAVI, which is comparatively well-defined for SAVR after nearly five decades of use (25, 26). The elevated incidence of long-term secondary outcomes among TAVI patients such as PPM implantation, PVL, and moderate to severe PPM in comparison to SAVR, and the inferiority of the latter in terms of major bleeding and new-onset atrial fibrillation as reported previously (10, 11, 13, 14, 25, 26, 44) is further re-enforced by our robust study results, adding weight to the argument for careful patient selection in aortic valve disease, rather than a non-individualised adherence to guidelines.

## Data Availability

The original contributions presented in the study are included in the article/Supplementary Material, further inquiries can be directed to the corresponding authors.
