# Supplementary material for: Evaluating long-term outcomes and the impact of small aortic annulus on valve replacement—a novel systematic review and meta-analysis comparing surgery vs. transcatheter interventions
Source: Front Cardiovasc Med. 2025 Jun 26;12:1555853. doi: 10.3389/fcvm.2025.1555853 (PMC12241089; doi:10.3389/fcvm.2025.1555853)

Supplementary Material

Systematic review and meta-analysis of transcatheter vs. surgical aortic valve replacement: a focus on long-term outcomes and small surgical annulus

This supplemental material has been provided by the authors to give readers additional information about their work.

Supplementary Table 1: Search Strategy for MEDLINE

Search	Query
#1	Transcatheter aortic valve replacement[MeSH]
#2	TAVI
#3	TAVR
#4	Transcatheter aortic valve replacement
#5	Aortic valve replacement
#6	#1 OR #2 OR #3 OR #4 OR #5
#7	Heart valve prosthesis implantation[MeSH]
#8	SAVR
#9	Sternotomy
#10	Surgical aortic valve replacement
#11	#7 OR #8 OR #9 OR #10
#12	#6 AND #11
#13	Randomized controlled trial[MeSH]
#14	Cohort
#15	#13 OR #14
#16	#12 AND #15

Supplementary Table 2: Clinical characteristics and outcomes of the included studies

Study Authors	NYHA Functional class III or IV, n		STS, mean ± SD		Mean aortic annulus diameter, mm		Minimal aortic annulus diameter, mm		Aortic annulus area, mm2		LVEF, %		Mean aortic gradient, mm Hg		Aortic valve area, cm2	
	TAVR	SAVR	TAVR	SAVR	TAVR	SAVR	TAVR	SAVR	TAVR	SAVR	TAVR	SAVR	TAVR	SAVR	TAVR	SAVR
Gleason 2018	338	348	7.3±3.0	7.5±3.2	-	-	-	-	-	-	56.9 ± 12.5	56.0 ± 12.2	-	-	-	-
Deeb 2018	338	348	7.3±3.0	7.5±3.2	-	-	-	-	-	-	56.9 ± 12.5	56.0 ± 12.2	51.03 ±16.73	51.46 ± 15.99	0.7	0.7
Mack 2023	155	108	1.9 ± 0.7	1.9 ± 0.6	-	-	-	-	473.5 ± 83.3	479.6 ± 87.6	65.7 ± 9.0	66.2 ± 8.6	49.4 ± 12.8	48.3 ± 11.8	0.8 ± 0.2	0.8 ± 0.2
Kamioka 2019	19	18	6.7 ± 3.0	6.8 ± 5.0	19.1 ± 0.7	19.1 ± 0.6	-	-	-	-	60.9 ± 7.8	60.6 ± 9.5	64.6 ± 20.1	58.7 ± 18.3	0.56 ± 0.11	0.58 ± 0.13
Forrest 2023	182	193	2.0 ± 0.7	1.9 ± 0.7	-	-	-	-	-	-	61.7 ± 7.9 vs.	61.9 ± 7.7	-	-	-	-
Clavel 2009	-	-	-	-	20.2 ± 2.0	20.0 ± 1.6 (SAVR-ST) and 20.1 ± 1.7 (SAVR-SL)	-	-	-	-	54 ± 16	55 ± 15 (SAVR-ST) and 56 ± 14 (SAVR-SL)	47 ± 17	42 ± 16 (SAVR-ST) and 43 ± 8 (SAVR-SL)	0.64±0.18	0.72±0.17
Makkar 2020	782	776	5.8±2.1	5.8±1.9	-		-	-	-	-	56.2 ±10.8	55.3 ±11.9	44.9 ± 13.4	44.5 ± 12.5	(0.7±0.2)	(0.7±0.2)
Nishigawa 2023	34	11	-		19.5±1.3	19.7±1.3	-	-	-	-	64.4 (60.6-66.3)	63.8 (60.6-65.8)	53±16.8	61.0±19.9	0.68 (0.56-0.77)	0.68 (0.54-0.74)
Van Meighem 2022	520	463	4.4 ± 1.5	4.5 ± 1.6	-	-	18	-	-	-	-	-	-	-	≤1.0 cm2	
Rodes-Cabau 2023	23	24	2.55 (1.79-3.27)	2.4 (1.7-3.3)	21.2 (20.5-22.0)	21.0 (20.4-22.0)	19.0 (17.7-19.9)	18.5 (17.3-19.6)	348±42	343±39	62±7	62±8	47±17	49±17	0.67±0.18	0.74±0.36

Dionne 2017	-	-	-	-	19.8±0.9	19.8±0.9	-	-	-	-	60±9	61±6	48±19	47±18	0.65±0.20	0.73±0.23
Guimaraes 2019	-	-	-	-	22.3±2.3	-	19.9 ±2.3	-	-	-	TAVR: 57 +/- 12 vs TAVR: 57 +/- 10, Balloon expanding valve: 55 +/- 12, self-expanding valve: 57 +/- 10		44 ± 18	42 ± 18	-	-
Rodes-Cabau 2014	425	436	11.5±2.9	11.9±3.2	-	-	-	-	-	-	56±12	55±12	42.7±14.7	45.8±14.3	0.64±0.18	0.60±0.20
Salna 2018	-	-	6.8 ± 1.3	4.0 ± 0.63	20.4 ± 1.4	21.0 ± 1.0	-	-	-	-	65 ± 3.25	55± 3.47	42.1 ± 15.5	47.4 ± 17.0	0.63 ± 0.21	0.67 ± 0.32

Supplementary Table 3: Risk of bias in observational studies via NOS

	Selection				Comparability		Outcome			
**Study**	**Representativeness of the exposed cohort**	**Selection of the non-exposed cohort**	**Ascertainment of exposure**	**Demonstration that outcome of interest was not present at the start of study**	**Controls for the most important risk factors**	**Controls for other risk factors**	**Assessment of outcome**	**Was follow up long enough for outcomes to occur**	**Adequacy of follow up of cohorts**	**Total quality score**
Rhodes-Cabau,2014	1	1	1	1	1	1	1	1	1	**9**
Nishigawa 2023	1	1	1	1	1	0	1	1	0	**7**
Kamioka 2019	1	1	1	0	0	1	1	1	0	**6**
Guimaraes 2019	1	1	1	1	1	1	1	1	1	**9**
Repossin,2017	1	1	1	1	1	1	1	1	1	**9**
Dionne, 2017	1	1	1	1	1	1	1	0	0	**7**
Clavel, 2009	1	1	1	1	1	0	1	1	1	**8**
Salna, 2017	1	1	1	1	1	0	1	1	0	**7**

Deeb, 2018	1	1	1	1	1	0	1	1	1	**8**

Supplementary Figure 1. Risk of bias in RCTs

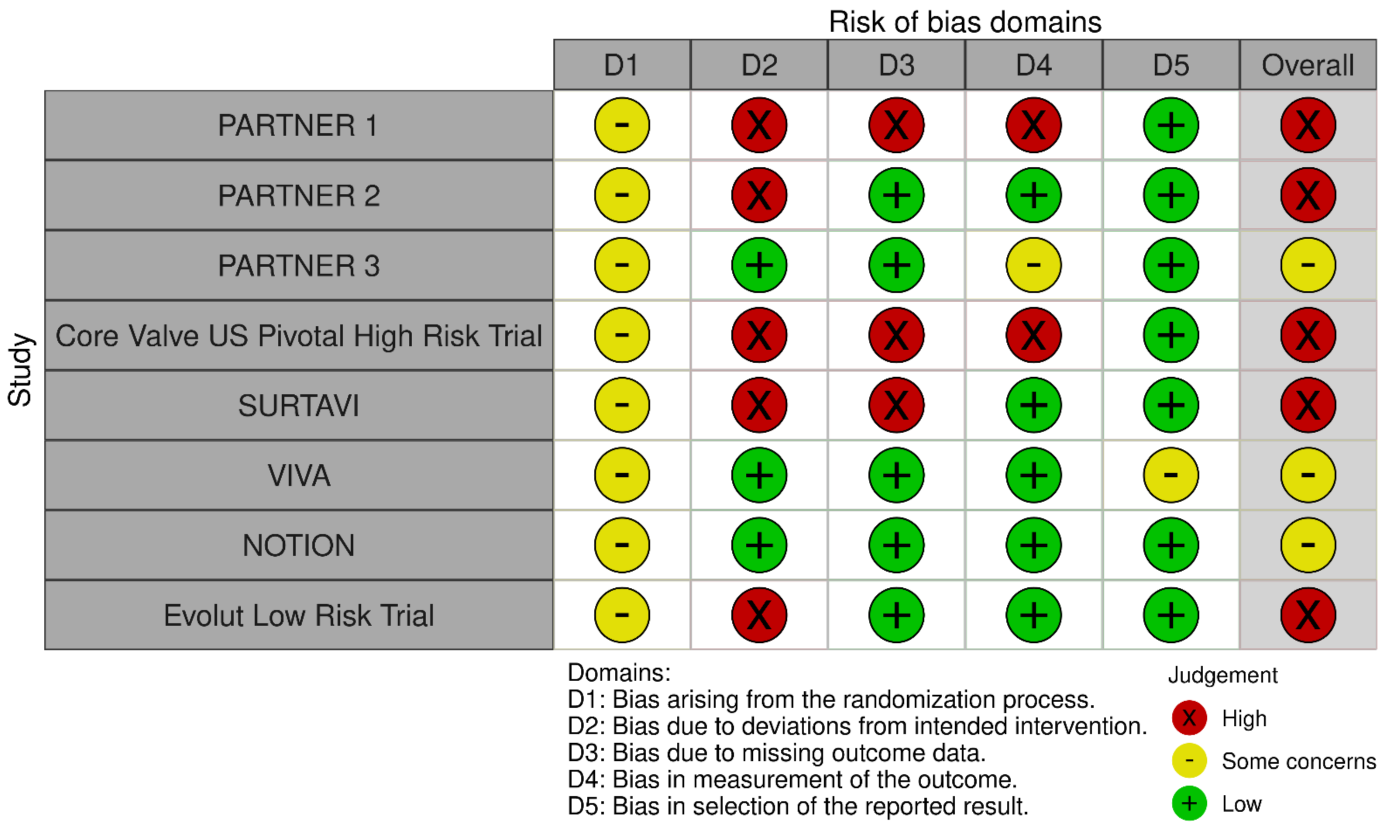
Risk of bias domains
D1
D2
D3
D4
D5
Overall
Study
PARTNER 1 - X X X + X
PARTNER 2 - X + + + X
PARTNER 3 - + + - + -
Core Valve US Pivotal High Risk Trial - X X X + X
SURTAVI - X X + + X
VIVA - + + + - -
NOTION - + + + + -
Evolut Low Risk Trial - X + + + X
Domains:
D1: Bias arising from the randomization process.
D2: Bias due to deviations from intended intervention.
D3: Bias due to missing outcome data.
D4: Bias in measurement of the outcome.
D5: Bias in selection of the reported result.
Judgement
X High
- Some concerns
+ Low

Supplementary Figure 2. Forest plot of cardiovascular mortality at 3 to 5-year follow-up (TAVI vs SAVR)

Study or Subgroup	TAVI Events	TAVI Total	SAVR Events	SAVR Total	Weight	Risk Ratio IV, Random, 95% CI
Forrest 2023	29	704	36	624	3.5%	0.71 [0.44, 1.15]
Gleason 2018	134	391	115	359	19.4%	1.07 [0.87, 1.31]
Mack 2015	147	348	123	351	22.8%	1.21 [1.00, 1.45]
Mack 2023	26	496	21	454	2.6%	1.13 [0.65, 1.99]
Makkar 2020	245	1011	223	1021	31.8%	1.11 [0.95, 1.30]
Thyregod 2019	30	145	31	135	4.1%	0.90 [0.58, 1.40]
Van Mieghem 2022	136	764	116	667	15.9%	1.02 [0.82, 1.28]
**Total (95% CI)**		**3859**		**3611**	**100.0%**	**1.08 [0.99, 1.18]**
Total events	747		665			

Heterogeneity: Tau² = 0.00; Chi² = 5.20, df = 6 (P = 0.52); I² = 0%
Test for overall effect: Z = 1.73 (P = 0.08)

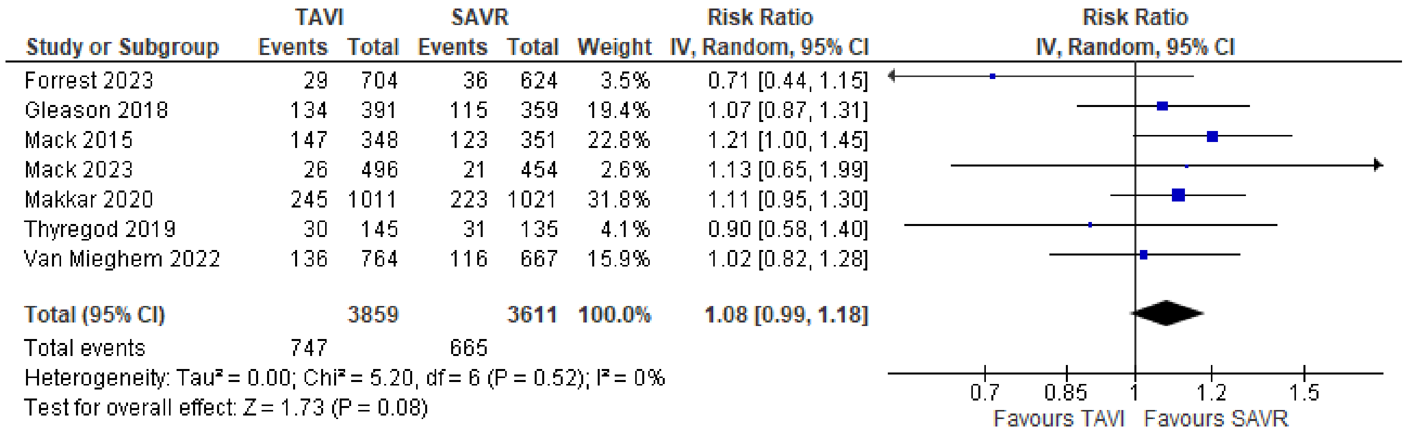

Supplementary Figure 3. Forest plot of disabling stroke at 3 to 5-year follow-up (TAVI vs SAVR)

Study or Subgroup	TAVI Events	TAVI Total	SAVR Events	SAVR Total	Weight	Risk Ratio IV, Random, 95% CI
Forrest 2023	16	704	22	624	10.3%	0.64 [0.34, 1.22]
Gleason 2018	38	391	38	359	22.0%	0.92 [0.60, 1.41]
Mack 2023	13	496	11	454	6.7%	1.08 [0.49, 2.39]
Makkar 2020	83	1011	75	1021	41.6%	1.12 [0.83, 1.51]
Van Mieghem 2022	31	756	40	690	19.3%	0.71 [0.45, 1.12]
**Total (95% CI)**		**3358**		**3148**	**100.0%**	**0.92 [0.75, 1.14]**
Total events	181		186			

Heterogeneity: Tau² = 0.00; Chi² = 4.24, df = 4 (P = 0.37); I² = 6%
Test for overall effect: Z = 0.75 (P = 0.45)

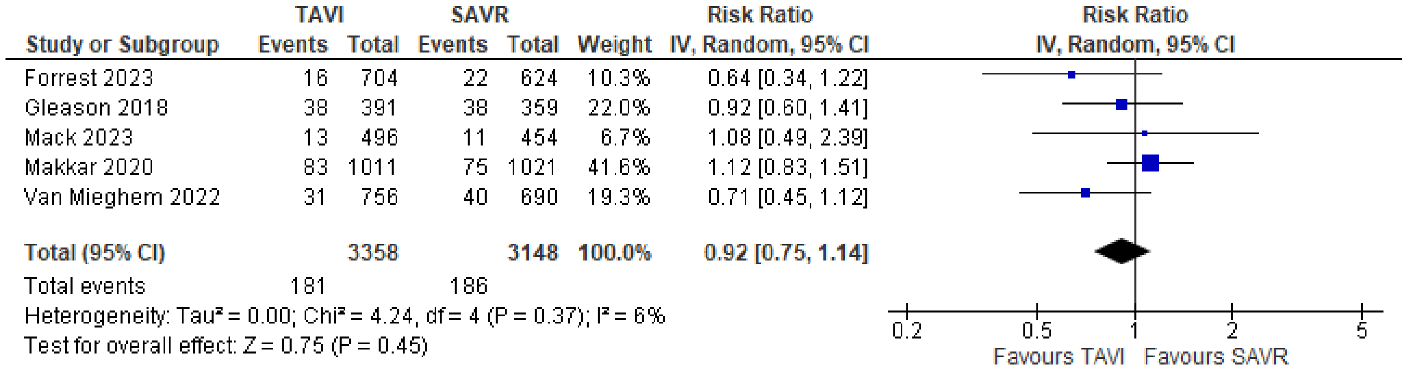

Supplementary Figure 4. Forest plot of death or disabling stroke at 3 to 5-year follow-up (TAVI vs SAVR)

Study or Subgroup	TAVI Events	TAVI Total	SAVR Events	SAVR Total	Weight	Risk Ratio IV, Random, 95% CI
Forrest 2023	53	704	67	624	6.2%	0.70 [0.50, 0.99]
Gleason 2018	216	391	193	359	19.6%	1.03 [0.90, 1.17]
Mack 2015	236	348	200	351	21.2%	1.19 [1.06, 1.34]
Mack 2023	55	496	41	454	5.2%	1.23 [0.84, 1.80]
Makkar 2020	456	1011	388	1021	22.6%	1.19 [1.07, 1.32]
Thyregod 2019	55	145	49	135	7.5%	1.05 [0.77, 1.42]
Van Mieghem 2022	255	815	217	705	17.6%	1.02 [0.87, 1.18]
**Total (95% CI)**		**3910**		**3649**	**100.0%**	**1.08 [0.98, 1.19]**
Total events	1326		1155			

Heterogeneity: Tau² = 0.01; Chi² = 12.90, df = 6 (P = 0.04); I² = 53%
Test for overall effect: Z = 1.54 (P = 0.12)

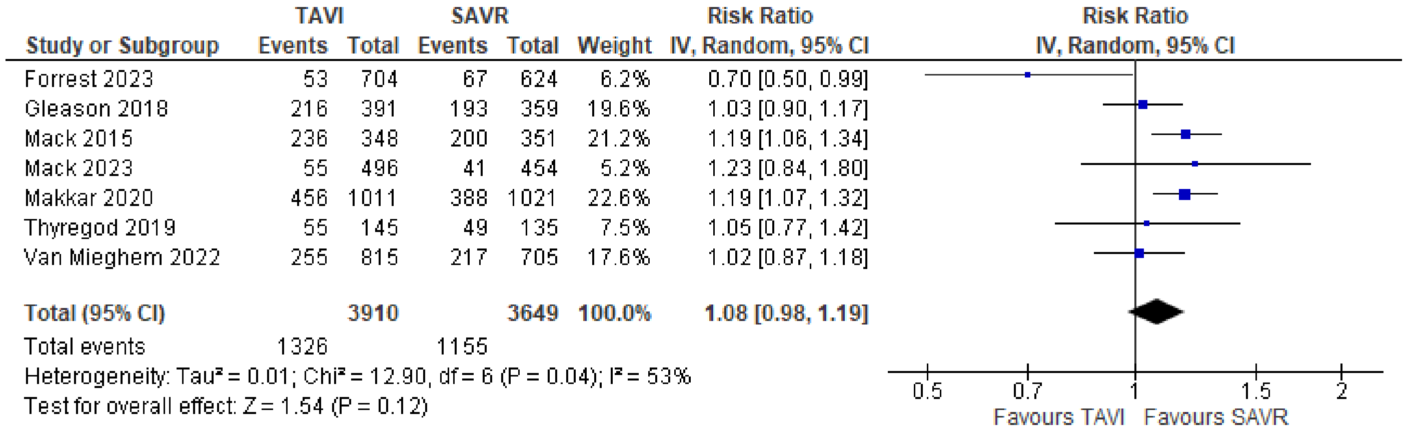

Supplementary Figure 5. Forest plot of death, stroke, or hospitalization at 3 to 5-year follow-up (TAVI vs SAVR)

Study or Subgroup	TAVI Events	TAVI Total	SAVR Events	SAVR Total	Weight	Risk Ratio IV, Random, 95% CI
Forrest 2023	95	704	110	624	16.9%	0.77 [0.59, 0.99]
Mack 2023	111	496	117	454	18.3%	0.87 [0.69, 1.09]
Makkar 2020	597	1011	490	1021	26.2%	1.23 [1.13, 1.34]
Thyregod 2019	55	145	49	135	14.3%	1.05 [0.77, 1.42]
Van Mieghem 2022	435	1568	353	1359	24.4%	1.07 [0.95, 1.20]
**Total (95% CI)**		**3924**		**3593**	**100.0%**	**1.01 [0.85, 1.19]**
Total events	1293		1119			

Heterogeneity: Tau² = 0.03; Chi² = 19.43, df = 4 (P = 0.0006); I² = 79%
Test for overall effect: Z = 0.07 (P = 0.95)

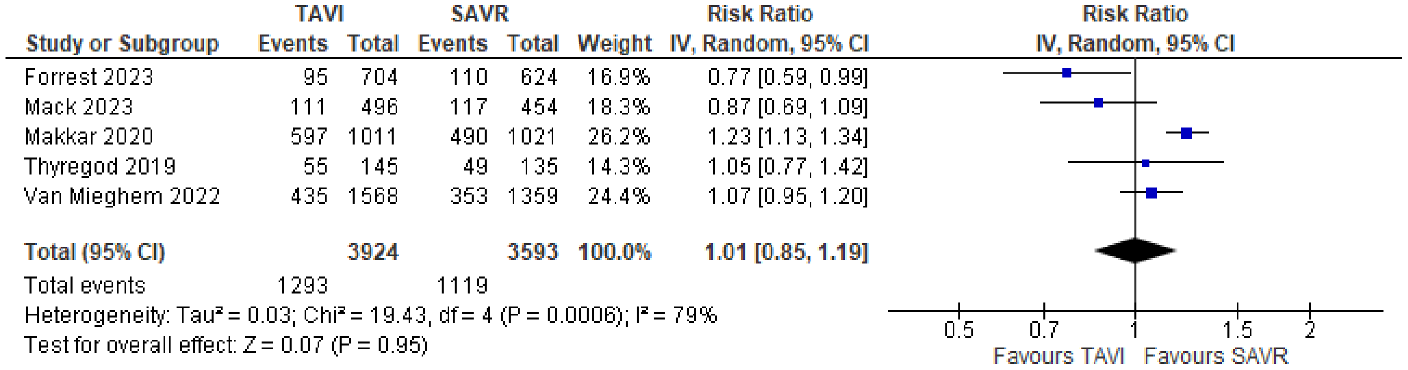

Supplementary Figure 6. Forest plot of major vascular complications at 3 to 5-year follow-up (TAVI vs SAVR)

	TAVI		SAVR			Risk Ratio
Study or Subgroup	Events	Total	Events	Total	Weight	IV, Random, 95% CI
Forrest 2023	30	704	25	624	36.2%	1.06 [0.63, 1.79]
Gleason 2018	27	391	7	359	29.2%	3.54 [1.56, 8.03]
Mack 2015	41	348	14	351	34.6%	2.95 [1.64, 5.32]
Mack 2023	0	0	0	0		Not estimable
Makkar 2020	0	0	0	0		Not estimable
Thyregod 2019	0	0	0	0		Not estimable
Van Mieghem 2022	0	0	0	0		Not estimable
**Total (95% CI)**		**1443**		**1334**	**100.0%**	**2.15 [0.99, 4.70]**
Total events	98		46			

Heterogeneity: Tau² = 0.37; Chi² = 9.17, df = 2 (P = 0.01); I² = 78%
Test for overall effect: Z = 1.93 (P = 0.05)

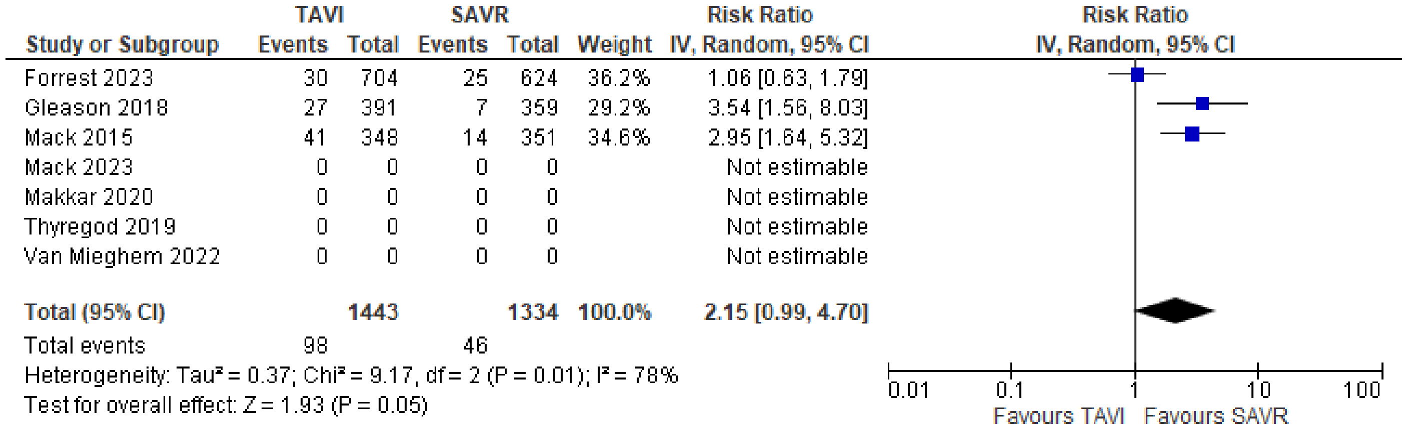

Supplementary Figure 7. Forest plot of major bleeding at 3 to 5-year follow-up (TAVI vs SAVR)

	TAVI		SAVR			Risk Ratio
Study or Subgroup	Events	Total	Events	Total	Weight	IV, Random, 95% CI
Gleason 2018	132	391	144	359	55.0%	0.84 [0.70, 1.02]
Mack 2015	75	348	103	351	29.1%	0.73 [0.57, 0.95]
Mack 2023	49	496	64	454	15.9%	0.70 [0.49, 0.99]
**Total (95% CI)**		**1235**		**1164**	**100.0%**	**0.79 [0.68, 0.90]**
Total events	256		311			

Heterogeneity: Tau² = 0.00; Chi² = 1.19, df = 2 (P = 0.55); I² = 0%
Test for overall effect: Z = 3.39 (P = 0.0007)

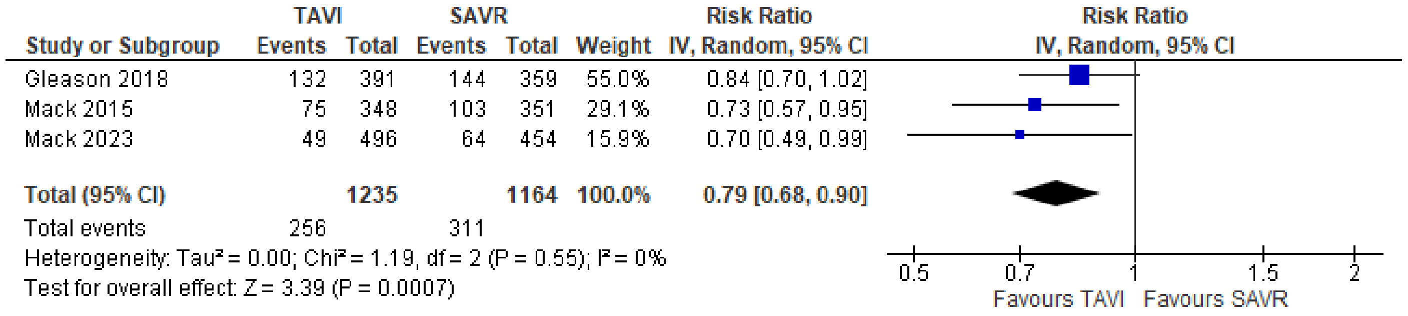

Supplementary Figure 8. Forest plot of MI at 3 to 5-year follow-up (TAVI vs SAVR)

	TAVI		SAVR			Risk Ratio
**Study or Subgroup**	**Events**	**Total**	**Events**	**Total**	**Weight**	**IV, Random, 95% CI**
Forrest 2023	24	704	15	624	14.1%	1.42 [0.75, 2.68]
Gleason 2018	10	391	9	359	8.5%	1.02 [0.42, 2.48]
Mack 2015	5	348	11	351	6.5%	0.46 [0.16, 1.31]
Mack 2023	10	496	18	454	10.8%	0.51 [0.24, 1.09]
Makkar 2020	84	1011	62	1021	29.1%	1.37 [1.00, 1.88]
Thyregod 2019	11	145	10	135	9.6%	1.02 [0.45, 2.33]
Van Mieghem 2022	45	726	30	556	21.5%	1.15 [0.73, 1.80]
**Total (95% CI)**		**3821**		**3500**	**100.0%**	**1.05 [0.79, 1.40]**
Total events	189		155			

Heterogeneity: Tau² = 0.05; Chi² = 9.07, df = 6 (P = 0.17); I² = 34%
Test for overall effect: Z = 0.35 (P = 0.73)

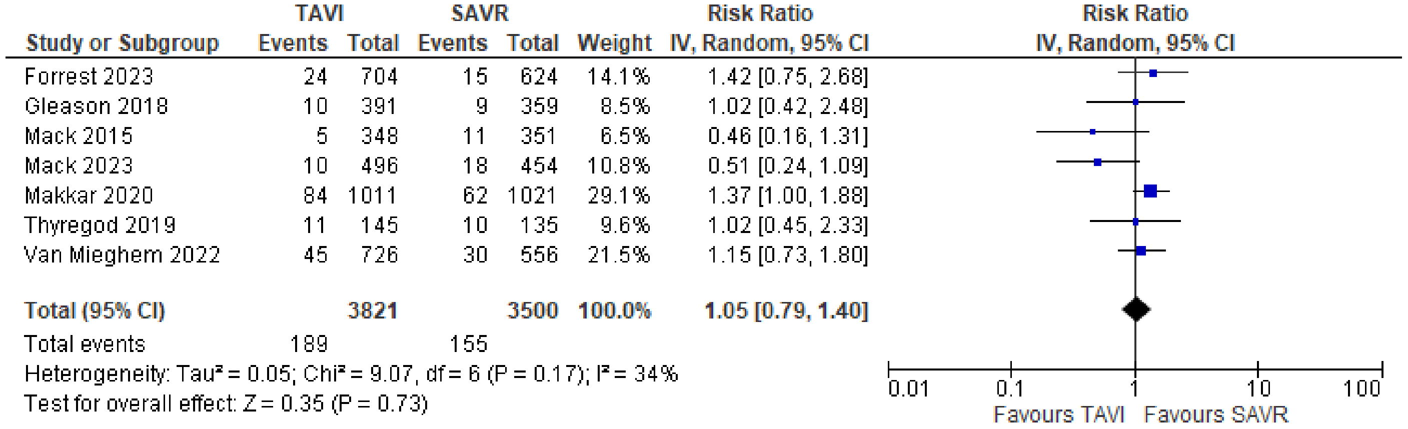

Supplementary Figure 9. Forest plot of infective endocarditis at 3 to 5-year follow-up (TAVI vs SAVR)

Study or Subgroup	TAVI Events	TAVI Total	SAVR Events	SAVR Total	Weight	Risk Ratio IV, Random, 95% CI
Forrest 2023	5	704	8	624	12.2%	0.55 [0.18, 1.68]
Gleason 2018	5	391	5	359	10.6%	0.92 [0.27, 3.15]
Mack 2015	5	348	6	351	11.3%	0.84 [0.26, 2.73]
Mack 2023	6	496	8	454	13.1%	0.69 [0.24, 1.96]
Makkar 2020	30	1011	19	1021	23.7%	1.59 [0.90, 2.81]
Thyregod 2019	9	145	6	135	13.8%	1.40 [0.51, 3.82]
Van Mieghem 2022	7	700	12	389	15.3%	0.32 [0.13, 0.82]
**Total (95% CI)**		**3795**		**3333**	**100.0%**	**0.85 [0.53, 1.37]**
Total events	67		64			

Heterogeneity: Tau² = 0.17; Chi² = 10.27, df = 6 (P = 0.11); I² = 42%
Test for overall effect: Z = 0.68 (P = 0.50)

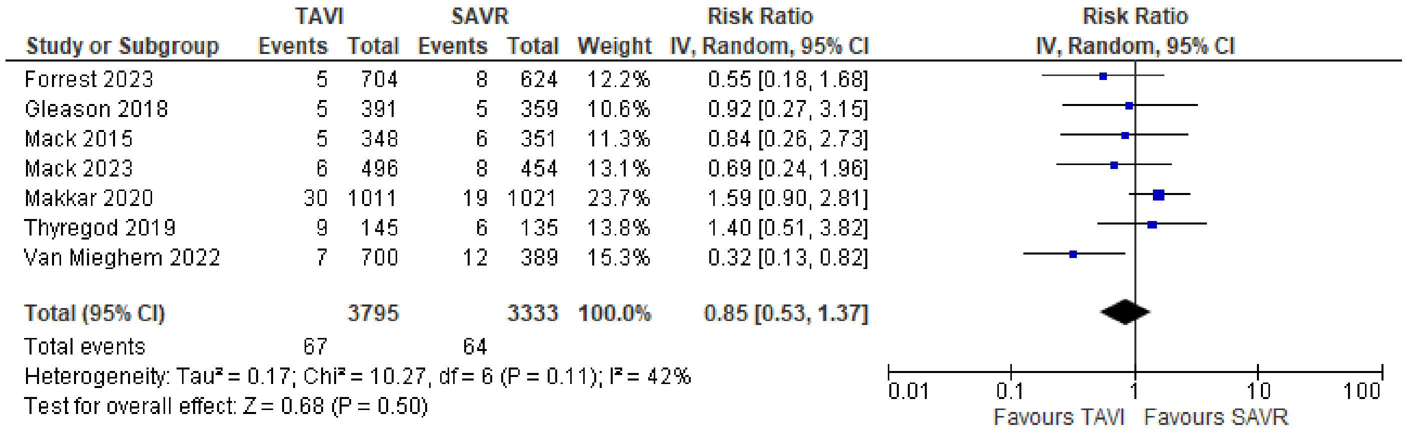

Supplementary Figure 10. Forest plot of atrial fibrillation at 3 to 5-year follow-up (TAVI vs SAVR)

Study or Subgroup	TAVI Events	TAVI Total	SAVR Events	SAVR Total	Weight	Risk Ratio IV, Random, 95% CI
Forrest 2023	94	704	271	624	27.0%	0.31 [0.25, 0.38]
Mack 2023	55	496	155	454	23.4%	0.32 [0.25, 0.43]
Makkar 2020	141	1011	291	1021	28.4%	0.49 [0.41, 0.59]
Thyregod 2019	34	145	82	135	21.2%	0.39 [0.28, 0.53]
**Total (95% CI)**		**2356**		**2234**	**100.0%**	**0.37 [0.29, 0.48]**
Total events	324		799			

Heterogeneity: Tau² = 0.05; Chi² = 12.61, df = 3 (P = 0.006); I² = 76%
Test for overall effect: Z = 7.85 (P < 0.00001)

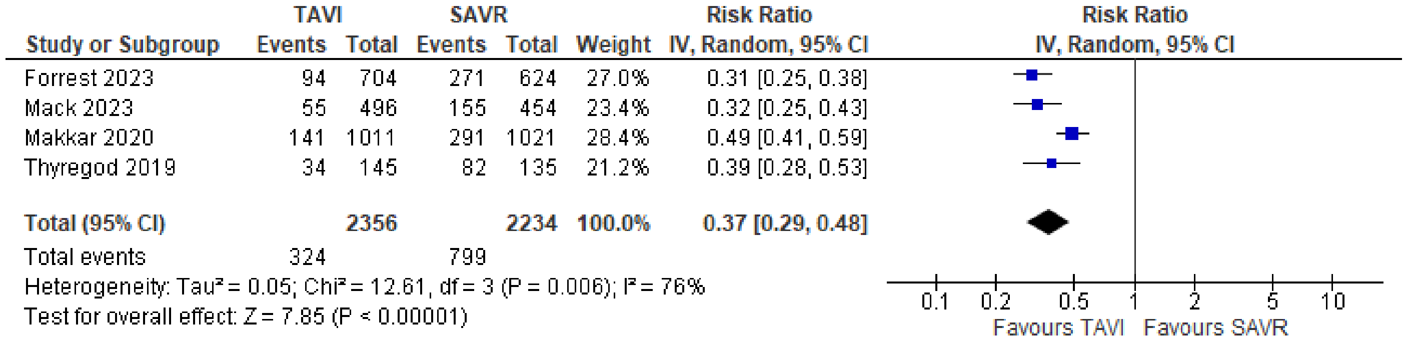

Supplementary Figure 11. Forest plot of rehospitalization at 3 to 5-year follow-up (TAVI vs SAVR)

Study or Subgroup	TAVI Events	TAVI Total	SAVR Events	SAVR Total	Weight	Risk Ratio IV, Random, 95% CI
Forrest 2023	52	704	59	624	15.4%	0.78 [0.55, 1.12]
Mack 2015	108	348	81	351	20.2%	1.34 [1.05, 1.72]
Mack 2023	65	496	74	454	17.4%	0.80 [0.59, 1.09]
Makkar 2020	281	1011	209	1021	24.4%	1.36 [1.16, 1.59]
Van Mieghem 2022	180	753	136	654	22.6%	1.15 [0.94, 1.40]
**Total (95% CI)**		**3312**		**3104**	**100.0%**	**1.09 [0.89, 1.35]**
Total events	686		559			

Heterogeneity: Tau² = 0.04; Chi² = 15.33, df = 4 (P = 0.004); I² = 74%
Test for overall effect: Z = 0.85 (P = 0.40)

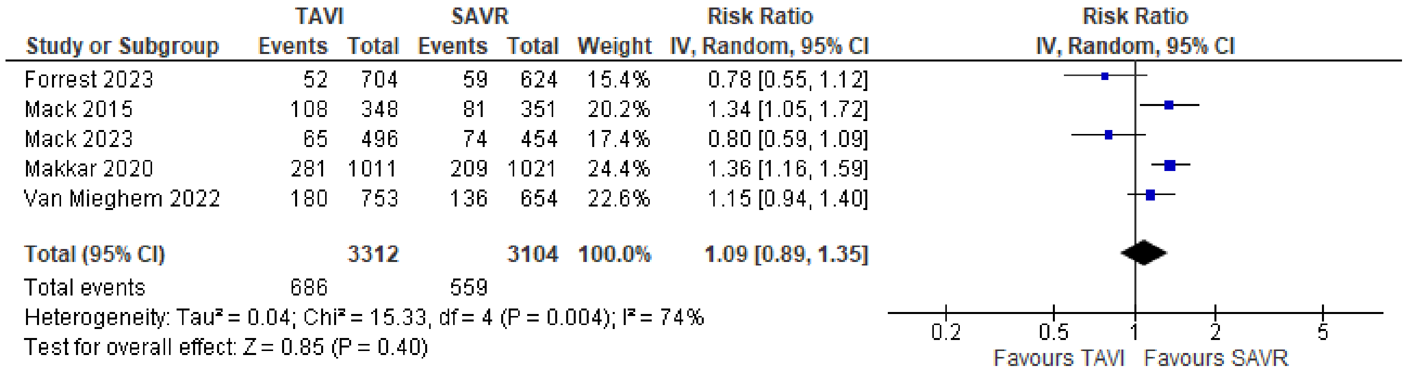

Supplementary Figure 12. Forest plot of re-intervention at 3 to 5-year follow-up (TAVI vs SAVR)

Study or Subgroup	TAVI Events	TAVI Total	SAVR Events	SAVR Total	Weight	Risk Ratio IV, Random, 95% CI
Forrest 2023	7	704	6	624	14.6%	1.03 [0.35, 3.06]
Gleason 2018	10	391	2	359	7.5%	4.59 [1.01, 20.81]
Mack 2023	12	496	12	454	27.6%	0.92 [0.42, 2.02]
Makkar 2020	6	1011	4	1021	10.8%	1.51 [0.43, 5.35]
Thyregod 2019	3	145	1	135	3.4%	2.79 [0.29, 26.53]
Van Mieghem 2022	27	771	11	579	35.9%	1.84 [0.92, 3.69]
**Total (95% CI)**		**3518**		**3172**	**100.0%**	**1.48 [0.98, 2.25]**
Total events	65		36			

Heterogeneity: Tau² = 0.00; Chi² = 4.69, df = 5 (P = 0.46); I² = 0%
Test for overall effect: Z = 1.87 (P = 0.06)

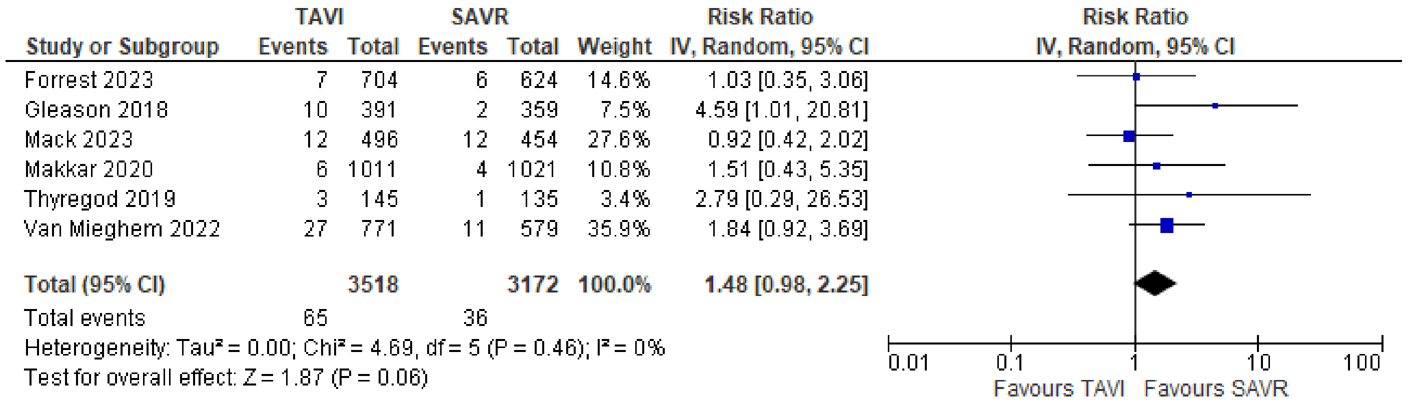

Supplementary Figure 13. Forest plot of pacemaker implantation at 3 to 5-year follow-up (TAVI vs SAVR)

Study or Subgroup	TAVI Events	TAVI Total	SAVR Events	SAVR Total	Weight	Risk Ratio IV, Random, 95% CI
Forrest 2023	162	704	58	624	15.6%	2.48 [1.87, 3.28]
Gleason 2018	105	391	50	359	15.2%	1.93 [1.42, 2.62]
Mack 2015	28	348	23	351	11.8%	1.23 [0.72, 2.09]
Mack 2023	63	496	43	454	14.3%	1.34 [0.93, 1.93]
Makkar 2020	138	1011	113	1021	16.2%	1.23 [0.98, 1.56]
Thyregod 2019	58	145	10	135	10.4%	5.40 [2.88, 10.13]
Van Mieghem 2022	289	739	94	623	16.5%	2.59 [2.11, 3.19]
**Total (95% CI)**		**3834**		**3567**	**100.0%**	**1.96 [1.43, 2.69]**
Total events	843		391			

Heterogeneity: Tau² = 0.15; Chi² = 41.90, df = 6 (P < 0.00001); I² = 86%
Test for overall effect: Z = 4.16 (P < 0.0001)

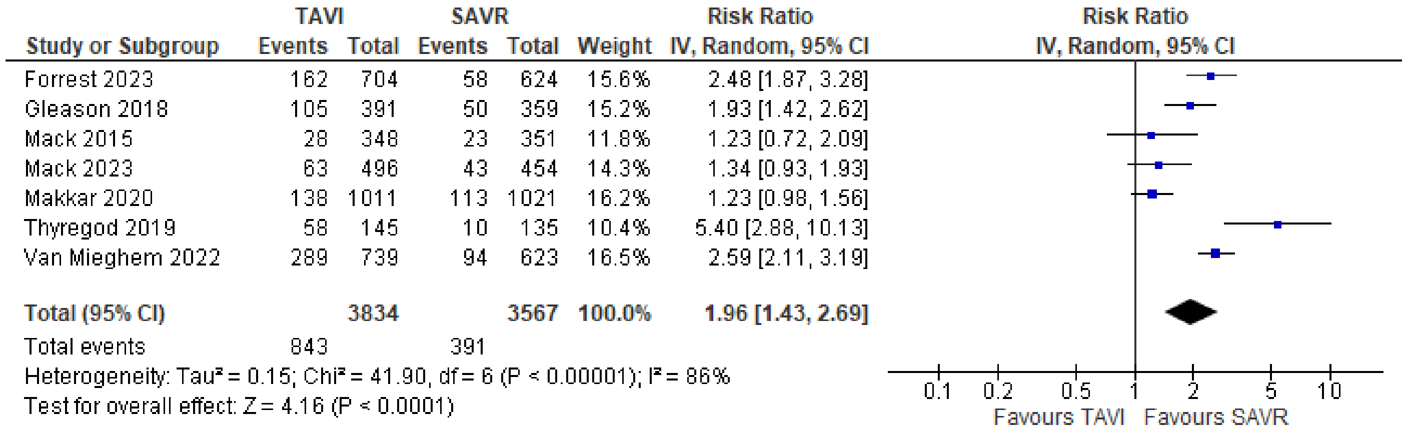

Supplementary Figure 14. Forest plot of cardiovascular mortality in patients with SAA (TAVI vs SAVR)

Study or Subgroup	smTAVI Events	smTAVI Total	smSAVR Events	smSAVR Total	Weight	Risk Ratio IV, Random, 95% CI
Deeb 2018	16	104	10	174	52.4%	2.68 [1.26, 5.68]
Repossini 2017	5	142	1	142	8.8%	5.00 [0.59, 42.26]
Rodés-Cabau 2014	10	98	7	83	38.8%	1.21 [0.48, 3.04]
**Total (95% CI)**		**344**		**399**	**100.0%**	**2.08 [1.09, 3.98]**
Total events	31		18			

Heterogeneity: Tau² = 0.06; Chi² = 2.41, df = 2 (P = 0.30); I² = 17%
Test for overall effect: Z = 2.21 (P = 0.03)

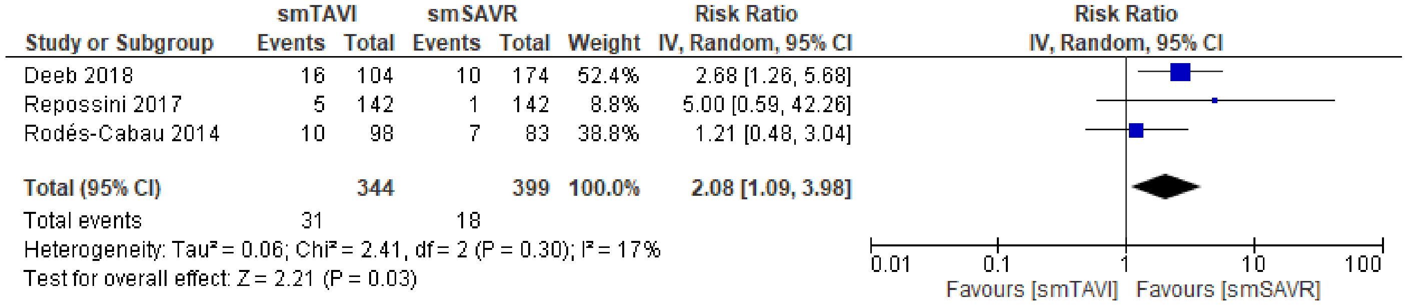

Supplementary Figure 15. Forest plot of disabling stroke in patients with SAA (TAVI vs SAVR)

Study or Subgroup	smTAVI Events	smTAVI Total	smSAVR Events	smSAVR Total	Weight	Risk Ratio IV, Random, 95% CI
Deeb 2018	5	104	4	74	74.1%	0.89 [0.25, 3.20]
Rodés-Cabau 2014	1	98	0	83	12.0%	2.55 [0.11, 61.66]
Rodés-Cabau 2023	0	77	3	74	14.0%	0.14 [0.01, 2.61]
**Total (95% CI)**		**279**		**231**	**100.0%**	**0.78 [0.26, 2.34]**
Total events	6		7			

Heterogeneity: Tau² = 0.00; Chi² = 1.90, df = 2 (P = 0.39); I² = 0%
Test for overall effect: Z = 0.45 (P = 0.65)

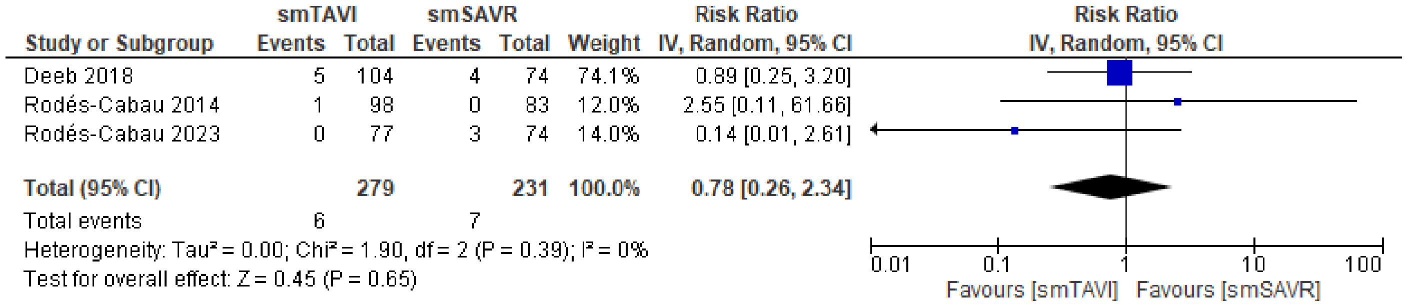

Supplementary Figure 16. Forest plot of death or disabling stroke in patients with SAA (TAVI vs SAVR)

Study or Subgroup	smTAVI Events	smTAVI Total	smSAVR Events	smSAVR Total	Weight	Risk Ratio IV, Random, 95% CI
Deeb 2018	24	104	20	74	52.9%	0.85 [0.51, 1.43]
Repossini 2017	9	142	3	142	18.8%	3.00 [0.83, 10.85]
Rodés-Cabau 2023	7	77	8	74	28.3%	0.84 [0.32, 2.20]
**Total (95% CI)**		**323**		**290**	**100.0%**	**1.08 [0.57, 2.03]**
Total events	40		31			

Heterogeneity: Tau² = 0.13; Chi² = 3.28, df = 2 (P = 0.19); I² = 39%
Test for overall effect: Z = 0.23 (P = 0.82)

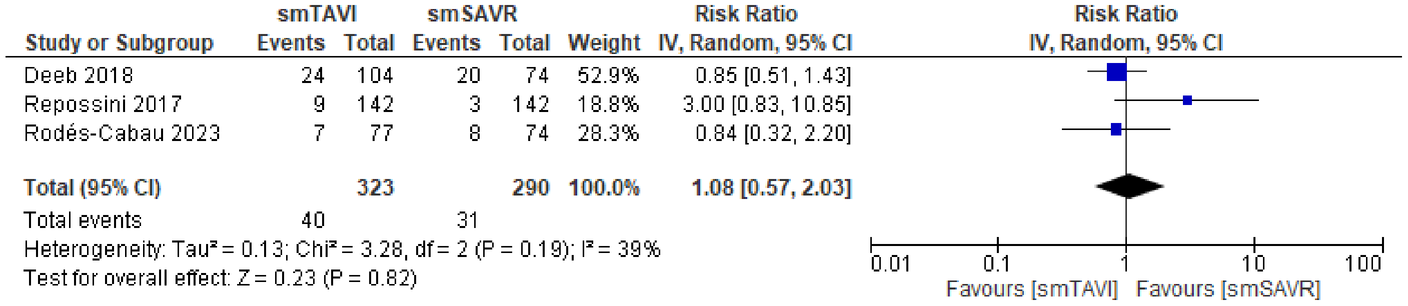

Supplementary Figure 17. Forest plot of death stroke or hospitalization in patients with SAA (TAVI vs SAVR)

Study or Subgroup	smTAVI Events	smTAVI Total	smSAVR Events	smSAVR Total	Weight	Risk Ratio IV, Random, 95% CI
Repossini 2017	9	142	3	142	37.2%	3.00 [0.83, 10.85]
Rodés-Cabau 2023	22	153	23	148	62.8%	0.93 [0.54, 1.59]
**Total (95% CI)**		**295**		**290**	**100.0%**	**1.43 [0.47, 4.37]**
Total events	31		26			

Heterogeneity: Tau² = 0.44; Chi² = 2.73, df = 1 (P = 0.10); I² = 63%
Test for overall effect: Z = 0.63 (P = 0.53)

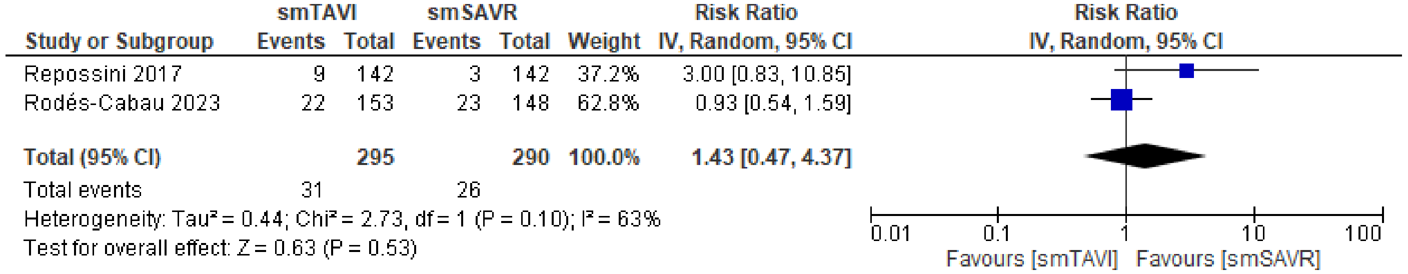

Supplementary Figure 18. Forest plot of major vascular complications in patients with SAA (TAVI vs SAVR)

Study or Subgroup	smTAVI Events	smTAVI Total	smSAVR Events	smSAVR Total	Weight	Risk Ratio IV, Random, 95% CI
Deeb 2018	10	104	0	74	14.1%	15.00 [0.89, 252.04]
Kamioka 2019	1	35	2	59	18.6%	0.84 [0.08, 8.96]
Repossini 2017	10	142	0	142	14.1%	21.00 [1.24, 354.98]
Rodés-Cabau 2014	18	98	6	83	53.1%	2.54 [1.06, 6.10]
**Total (95% CI)**		**379**		**358**	**100.0%**	**3.58 [1.10, 11.61]**
Total events	39		8			

Heterogeneity: Tau² = 0.48; Chi² = 4.32, df = 3 (P = 0.23); I² = 30%
Test for overall effect: Z = 2.12 (P = 0.03)

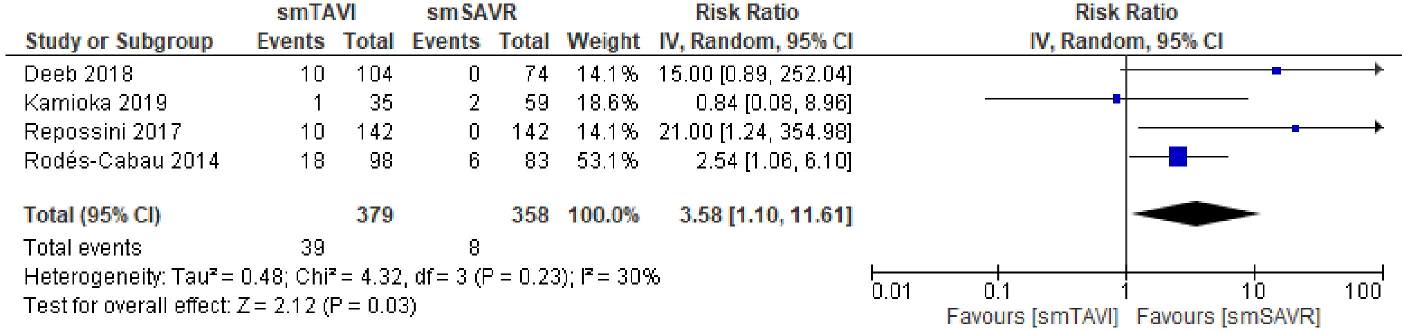

Supplementary Figure 19. Forest plot of major bleeding in patients with SAA (TAVI vs SAVR)

Study or Subgroup	smTAVI Events	smTAVI Total	smSAVR Events	smSAVR Total	Weight	Risk Ratio IV, Random, 95% CI
Deeb 2018	57	104	59	74	24.2%	0.69 [0.56, 0.85]
Kamioka 2019	15	35	10	59	16.4%	2.53 [1.28, 5.00]
Repossini 2017	49	142	36	142	22.1%	1.36 [0.95, 1.95]
Rodés-Cabau 2014	17	98	25	83	18.9%	0.58 [0.33, 0.99]
Rodés-Cabau 2023	10	77	18	74	16.1%	0.53 [0.26, 1.08]
Salna 2018	0	40	3	130	2.3%	0.46 [0.02, 8.65]
**Total (95% CI)**		**496**		**562**	**100.0%**	**0.91 [0.57, 1.45]**
Total events	148		151			

Heterogeneity: Tau² = 0.22; Chi² = 23.94, df = 5 (P = 0.0002); I² = 79%
Test for overall effect: Z = 0.39 (P = 0.69)

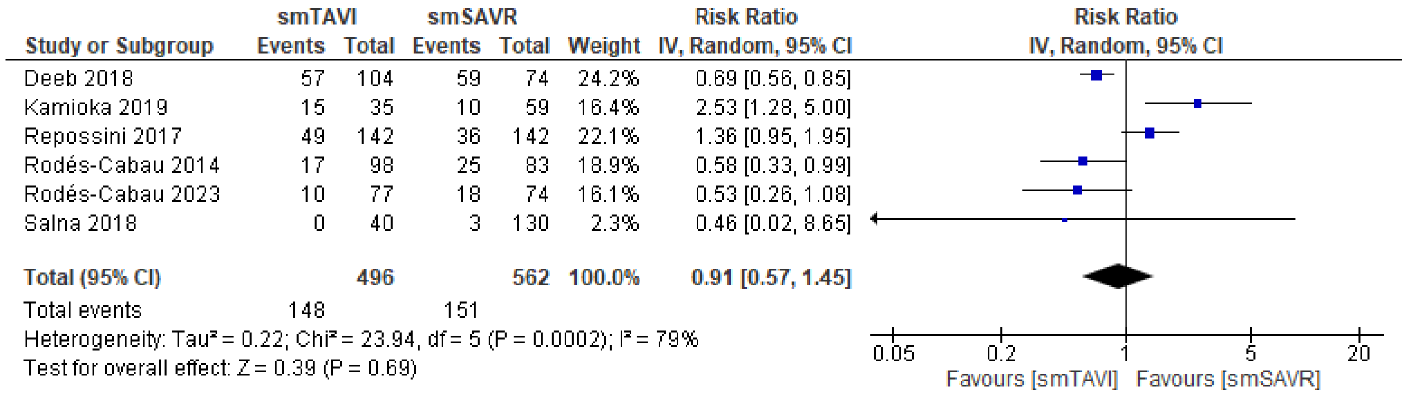

Supplementary Figure 20. Forest plot of MI in patients with SAA (TAVI vs SAVR)

Study or Subgroup	smTAVI Events	smTAVI Total	smSAVR Events	smSAVR Total	Weight	Risk Ratio IV, Random, 95% CI
Repossini 2017	0	142	0	142		Not estimable
Rodés-Cabau 2014	0	98	2	83	25.3%	0.17 [0.01, 3.49]
Rodés-Cabau 2023	2	77	3	74	74.7%	0.64 [0.11, 3.73]
Salna 2018	0	40	0	130		Not estimable
**Total (95% CI)**		**357**		**429**	**100.0%**	**0.46 [0.10, 2.09]**
Total events	2		5			

Heterogeneity: Tau² = 0.00; Chi² = 0.55, df = 1 (P = 0.46); I² = 0%
Test for overall effect: Z = 1.01 (P = 0.31)

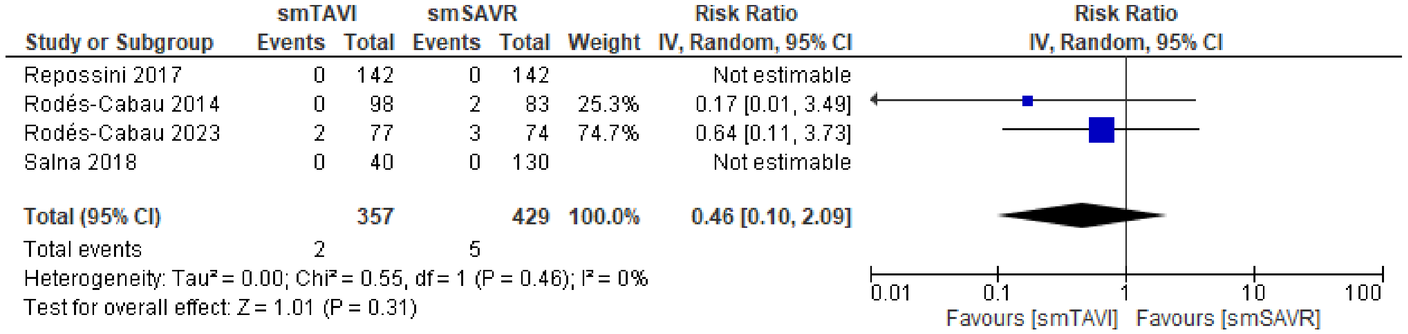

Supplementary Figure 21. Forest plot of LOS in ICU in patients with SAA (TAVI vs SAVR)

Study or Subgroup	smTAVI Mean	smTAVI SD	smTAVI Total	smSAVR Mean	smSAVR SD	smSAVR Total	Weight	Mean Difference IV, Random, 95% CI
Repossini 2017	3.2	2	142	1.6	1.8	142	52.1%	1.60 [1.16, 2.04]
Salna 2018	2.35	2.3	40	2.52	2.62	130	47.9%	-0.17 [-1.01, 0.67]
**Total (95% CI)**			**182**			**272**	**100.0%**	**0.75 [-0.98, 2.49]**

Heterogeneity: Tau² = 1.45; Chi² = 13.27, df = 1 (P = 0.0003); I² = 92%
Test for overall effect: Z = 0.85 (P = 0.39)

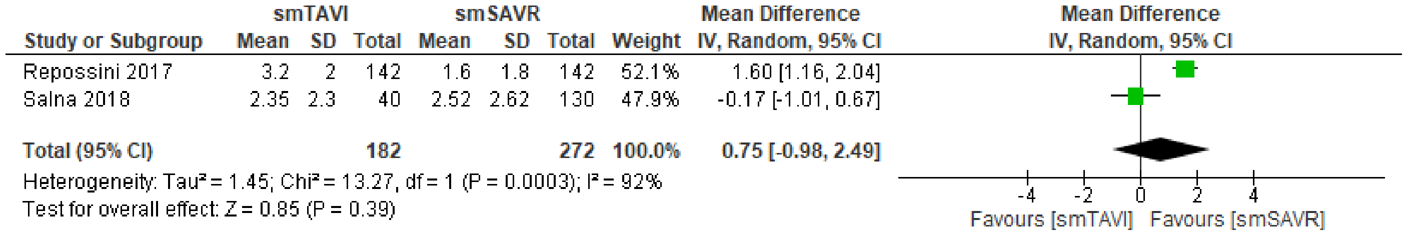

Supplementary Figure 22. Forest plot of LOS in hospital in patients with SAA (TAVI vs SAVR)

	smTAVI			smSAVR				Mean Difference
Study or Subgroup	Mean	SD	Total	Mean	SD	Total	Weight	IV, Random, 95% CI
Rodés-Cabau 2023	3	2.96	231	8	2.96	592	87.7%	-5.00 [-5.45, -4.55]
Salna 2018	6.4	4.6	40	10.4	6	130	12.3%	-4.00 [-5.76, -2.24]
**Total (95% CI)**			**271**			**722**	**100.0%**	**-4.88 [-5.52, -4.23]**

Heterogeneity: Tau² = 0.07; Chi² = 1.16, df = 1 (P = 0.28); I² = 14%
Test for overall effect: Z = 14.83 (P < 0.00001)

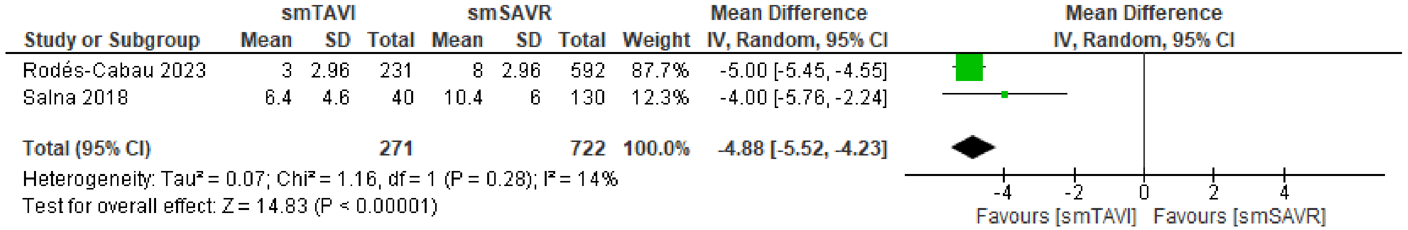

Supplementary Figure 23. Forest plot of rehospitalization in patients with SAA (TAVI vs SAVR)

	smTAVI		smSAVR			Risk Ratio
Study or Subgroup	Events	Total	Events	Total	Weight	IV, Random, 95% CI
Deeb 2018	23	104	14	74	48.4%	1.17 [0.65, 2.12]
Rodés-Cabau 2023	15	77	15	74	41.6%	0.96 [0.51, 1.82]
Salna 2018	3	40	7	130	10.0%	1.39 [0.38, 5.14]
**Total (95% CI)**		**221**		**278**	**100.0%**	**1.10 [0.73, 1.66]**
Total events	41		36			

Heterogeneity: Tau² = 0.00; Chi² = 0.34, df = 2 (P = 0.85); I² = 0%
Test for overall effect: Z = 0.44 (P = 0.66)

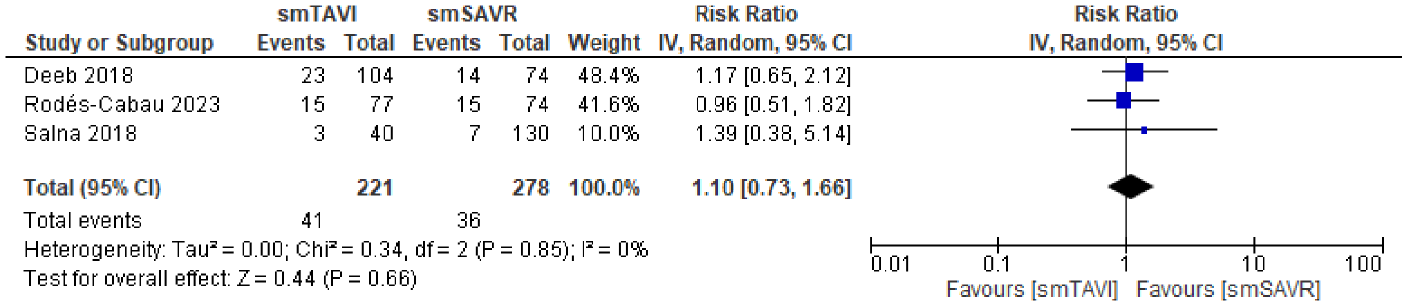

Supplementary Figure 24. Forest plot of re-intervention in patients with SAA (TAVI vs SAVR)

	smTAVI		smSAVR			Risk Ratio
Study or Subgroup	Events	Total	Events	Total	Weight	IV, Random, 95% CI
Deeb 2018	1	104	0	74	24.2%	2.14 [0.09, 51.88]
Nishigawa 2023	1	214	1	63	32.3%	0.29 [0.02, 4.64]
Rodés-Cabau 2023	2	77	1	74	43.4%	1.92 [0.18, 20.75]
**Total (95% CI)**		**395**		**211**	**100.0%**	**1.08 [0.22, 5.16]**
Total events	4		2			

Heterogeneity: Tau² = 0.00; Chi² = 1.26, df = 2 (P = 0.53); I² = 0%
Test for overall effect: Z = 0.09 (P = 0.93)

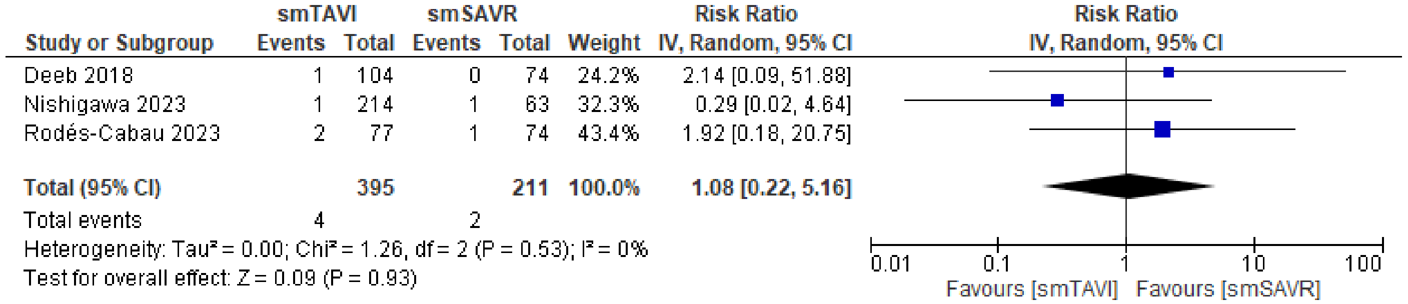

Supplementary Figure 25. Forest plot of PPM (moderate/severe) in patients with SAA (TAVI vs SAVR)

	smTAVI		smSAVR			Risk Ratio
Study or Subgroup	Events	Total	Events	Total	Weight	IV, Random, 95% CI
Clavel 2009	2	28	18	56	2.9%	0.22 [0.06, 0.89]
Deeb 2018	27	90	40	60	16.8%	0.45 [0.31, 0.65]
Dionne 2017	17	50	51	113	14.5%	0.75 [0.49, 1.17]
Guimarães 2019	192	357	284	357	24.8%	0.68 [0.61, 0.75]
Kamioka 2019	1	35	13	59	1.5%	0.13 [0.02, 0.95]
Nishigawa 2023	25	214	7	63	7.2%	1.05 [0.48, 2.32]
Repossini 2017	0	142	0	142		Not estimable
Rodés-Cabau 2014	32	98	22	83	13.9%	1.23 [0.78, 1.95]
Rodés-Cabau 2023	3	72	5	68	2.9%	0.57 [0.14, 2.28]
Salna 2018	18	40	38	69	15.5%	0.82 [0.55, 1.22]
**Total (95% CI)**		**1126**		**1070**	**100.0%**	**0.70 [0.54, 0.89]**
Total events	317		478			

Heterogeneity: Tau² = 0.06; Chi² = 18.86, df = 8 (P = 0.02); I² = 58%
Test for overall effect: Z = 2.84 (P = 0.005)

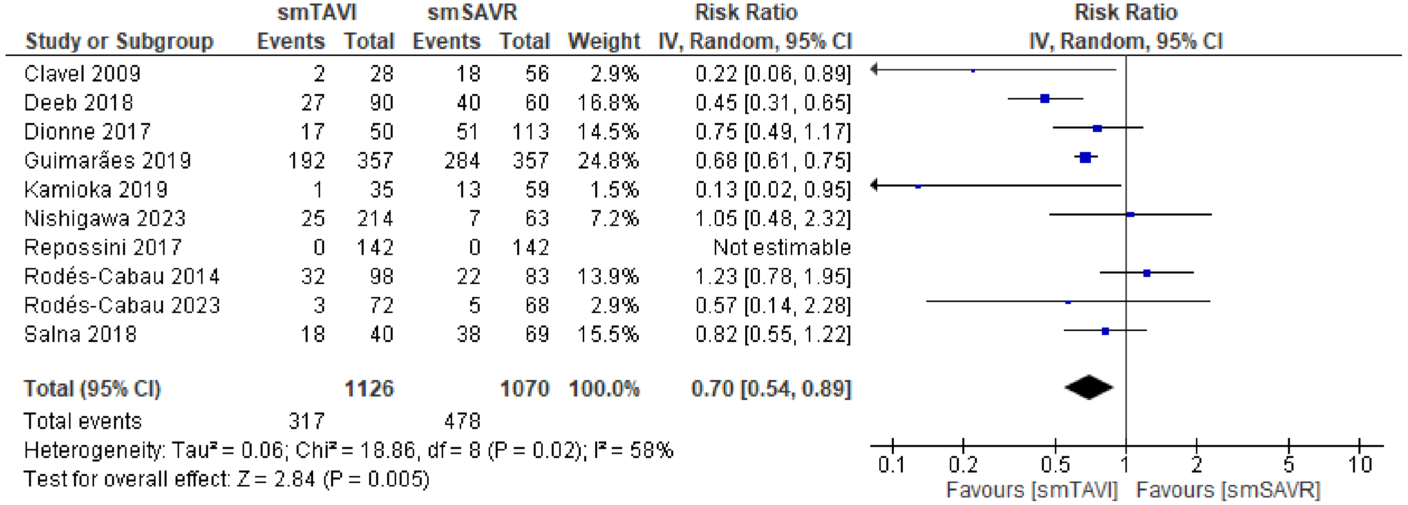

Supplementary Figure 26. Forest plot of pacemaker implantation in patients with SAA (TAVI vs SAVR)

	smTAVI		smSAVR			Risk Ratio
Study or Subgroup	Events	Total	Events	Total	Weight	IV, Random, 95% CI
Deeb 2018	24	104	7	74	40.8%	2.44 [1.11, 5.36]
Kamioka 2019	2	35	2	59	6.9%	1.69 [0.25, 11.44]
Nishigawa 2023	19	214	0	63	3.2%	11.61 [0.71, 189.62]
Repossini 2017	17	142	1	142	6.3%	17.00 [2.29, 126.03]
Rodés-Cabau 2023	11	77	5	74	24.9%	2.11 [0.77, 5.79]
Salna 2018	5	40	5	130	17.9%	3.25 [0.99, 10.66]
**Total (95% CI)**		**612**		**542**	**100.0%**	**2.87 [1.74, 4.75]**
Total events	78		20			

Heterogeneity: Tau² = 0.00; Chi² = 4.85, df = 5 (P = 0.43); I² = 0%
Test for overall effect: Z = 4.11 (P < 0.0001)

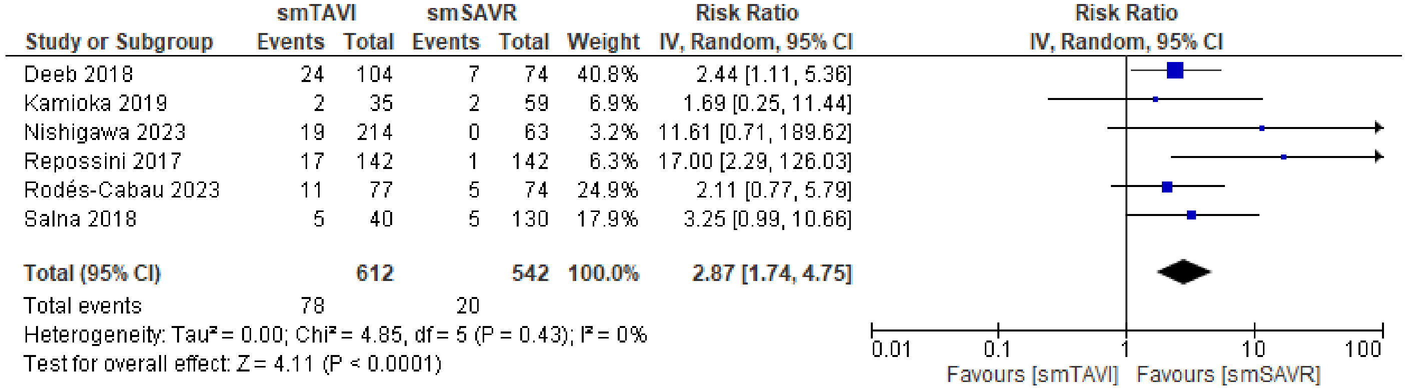

Supplementary Figure 27. Forest plot of effective orifice area (EOA) in patients with SAA (TAVI vs SAVR)

	smTAVI			smSAVR				Mean Difference
**Study or Subgroup**	**Mean**	**SD**	**Total**	**Mean**	**SD**	**Total**	**Weight**	**IV, Random, 95% CI**
Dionne 2017	1.5	0.55	50	1.45	0.34	113	11.6%	0.05 [-0.11, 0.21]
Guimarães 2019	1.46	0.39	357	1.25	0.37	357	17.8%	0.21 [0.15, 0.27]
Kamioka 2019	1.34	0.2	32	1.18	0.2	52	16.2%	0.16 [0.07, 0.25]
Repossini 2017	1.89	0.3	142	1.93	0.52	142	15.5%	-0.04 [-0.14, 0.06]
Rodés-Cabau 2014	1.49	0.51	98	1.23	0.4	83	13.5%	0.26 [0.13, 0.39]
Rodés-Cabau 2023	1.63	0.4	76	1.65	0.45	72	13.2%	-0.02 [-0.16, 0.12]
Salna 2018	1.35	0.34	40	1.28	0.49	69	12.1%	0.07 [-0.09, 0.23]
**Total (95% CI)**			**795**			**888**	**100.0%**	**0.10 [0.01, 0.19]**

Heterogeneity: Tau² = 0.01; Chi² = 29.27, df = 6 (P < 0.0001); I² = 80%
Test for overall effect: Z = 2.27 (P = 0.02)

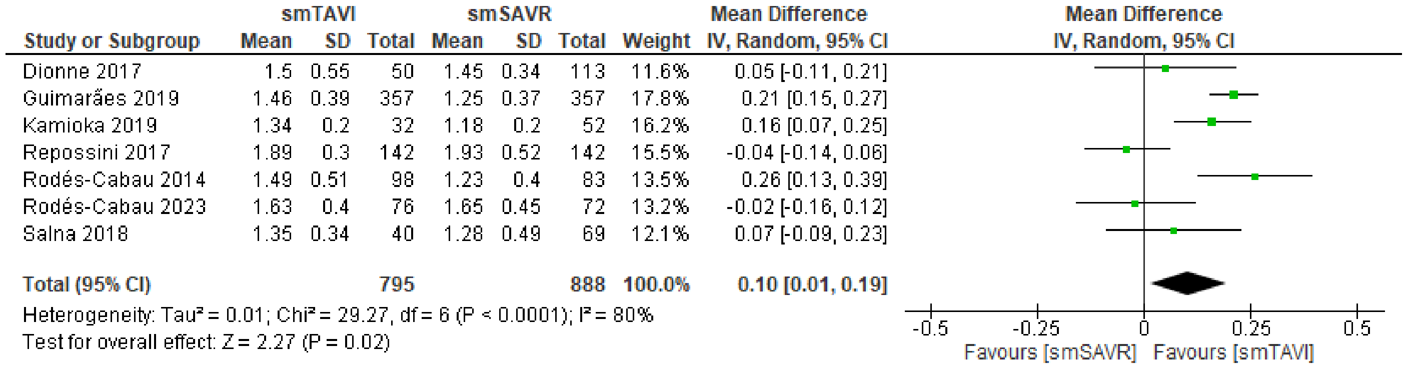

Supplementary Figure 28. Forest plot of EOAI in patients with SAA (TAVI vs SAVR)

	smTAVI			smSAVR				Mean Difference
**Study or Subgroup**	**Mean**	**SD**	**Total**	**Mean**	**SD**	**Total**	**Weight**	**IV, Random, 95% CI**
Clavel 2009	0.87	0.19	28	0.76	0.18	56	13.0%	0.11 [0.03, 0.19]
Dionne 2017	0.94	0.31	50	0.88	0.23	113	12.3%	0.06 [-0.04, 0.16]
Guimarães 2019	0.88	0.25	357	0.75	0.22	357	15.4%	0.13 [0.10, 0.16]
Nishigawa 2023	1.06	1.1132	214	1.03	0.5162	63	7.1%	0.03 [-0.17, 0.23]
Repossini 2017	1.02	0.31	142	1.12	0.22	142	14.2%	-0.10 [-0.16, -0.04]
Rodés-Cabau 2014	0.87	0.27	98	0.72	0.23	83	13.6%	0.15 [0.08, 0.22]
Rodés-Cabau 2023	0.99	0.28	76	0.98	0.27	72	12.7%	0.01 [-0.08, 0.10]
Salna 2018	0.87	0.23	40	0.78	0.33	69	11.7%	0.09 [-0.02, 0.20]
**Total (95% CI)**			**1005**			**955**	**100.0%**	**0.06 [-0.01, 0.13]**

Heterogeneity: Tau² = 0.01; Chi² = 46.76, df = 7 (P < 0.00001); I² = 85%
Test for overall effect: Z = 1.73 (P = 0.08)

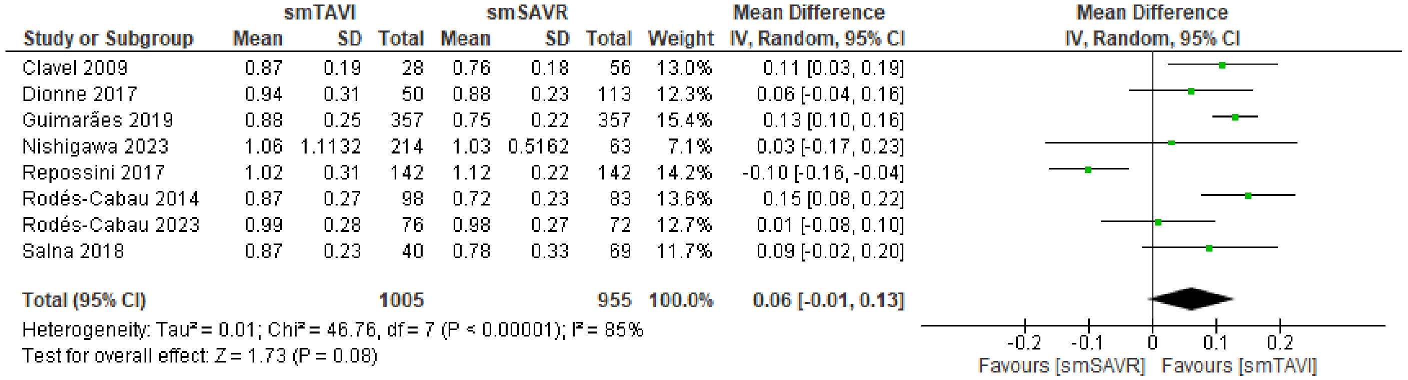

Supplementary Figure 29. Forest plot of PVL in patients with SAA (TAVI vs SAVR)

Study or Subgroup	smTAVI Events	smTAVI Total	smSAVR Events	smSAVR Total	Weight	Risk Ratio IV, Random, 95% CI
Deeb 2018	4	63	0	43	10.9%	6.19 [0.34, 112.05]
Dionne 2017	2	50	2	113	24.5%	2.26 [0.33, 15.59]
Guimarães 2019	9	357	0	357	11.3%	19.00 [1.11, 325.21]
Kamioka 2019	4	35	0	59	10.9%	15.00 [0.83, 270.54]
Nishigawa 2023	47	214	0	63	11.9%	28.28 [1.77, 452.36]
Repossini 2017	5	142	1	142	20.0%	5.00 [0.59, 42.26]
Rodés-Cabau 2014	3	98	0	83	10.5%	5.94 [0.31, 113.35]
Rodés-Cabau 2023	0	75	0	68		Not estimable
Salna 2018	0	40	0	69		Not estimable
**Total (95% CI)**		**1074**		**997**	**100.0%**	**6.91 [2.66, 17.97]**
Total events	74		3			

Heterogeneity: Tau² = 0.00; Chi² = 3.15, df = 6 (P = 0.79); I² = 0%

Test for overall effect: Z = 3.97 (P < 0.0001)

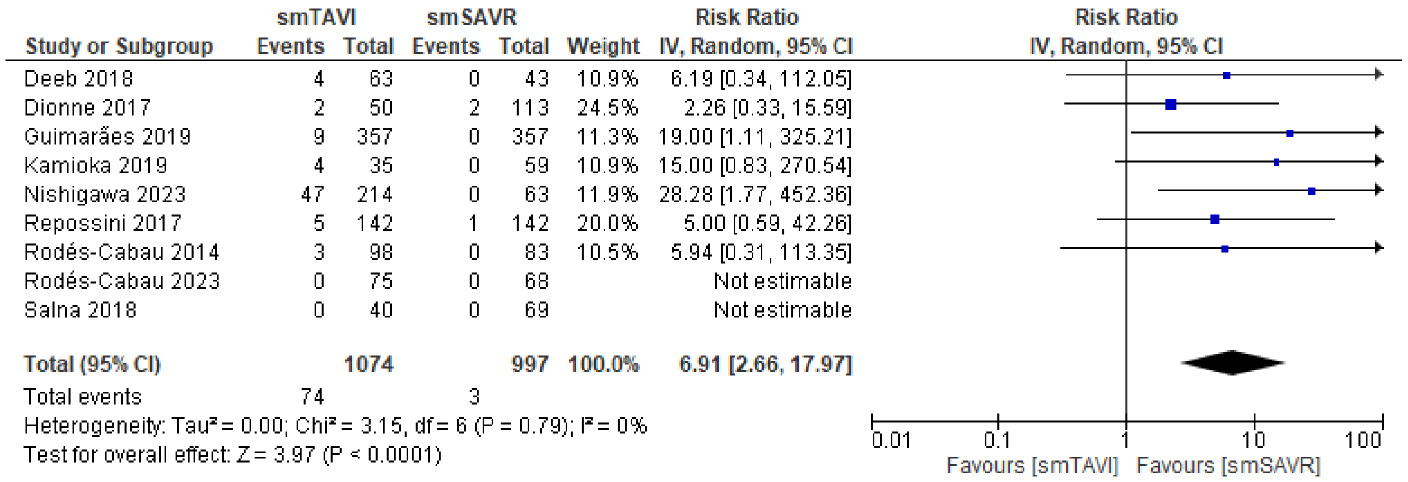

Supplement: Supplementary file 1 [file Datasheet1.pdf]
